# Supplementary material for: Community perceptions towards invasion of Prosopis juliflora, utilization, and its control options in Afar region, Northeast Ethiopia
Source: PLoS One. 2022 Jan 25;17(1):e0261838. doi: 10.1371/journal.pone.0261838 (PMC8789103; doi:10.1371/journal.pone.0261838)
Supplement: S1 Table — (DOCX) [file pone.0261838.s001.docx]

| Treatment variable | Type and definition | Measurement |
| --- | --- | --- |
| Invasion *P. juliflora* | Continuous, number before and after *P. juliflora* invasion | Numbers |
| Explanatory variable |  |  |
| Age | Continuous, age of household head | Years |
| Wealth | Ordinal for wealth class of household head | 1, 2, 3 if the household head is poor, medium and wealthy. |
| Sex | Dummy for sex of household head | 1 if the household head is male and 0 if female |
| Education | Ordinal for illiterate household head | 0,1,2,3 if the household head has no formal education, primary, secondary, and post-secondary education. |
| Position of households in the sites | Ordinal for member or chairman in sites | 0,1,2,3,4,5,6,7 if the household is non-positioned, positioned as chairman, treasurer, secretary, youth, committee member and member |
| Off/non-farm activities | Dummy, engagement of the household head in farm/off-farm/non-farm activities | 1, otherwise; 2, the household head is engaged in farm/off-farm/non-farm activities |

## Source: Shiferaw et al. [14]
