# Supplementary material for: Community perceptions towards invasion of Prosopis juliflora, utilization, and its control options in Afar region, Northeast Ethiopia
Source: PLoS One. 2022 Jan 25;17(1):e0261838. doi: 10.1371/journal.pone.0261838 (PMC8789103; doi:10.1371/journal.pone.0261838)
Supplement: S2 Table — (DOCX) [file pone.0261838.s002.docx]

| Response variables | | | | | | | | | | | | |  | |  | |  |  |  |
| --- | --- | --- | --- | --- | --- | --- | --- | --- | --- | --- | --- | --- | --- | --- | --- | --- | --- | --- | --- |
| Stat | MOL | | DHp | IYH | IIPWI | | WIn | | PSreg | | BPJ | | IPoNr | | DCmU | |  |  |  |
| *χ^2^* | 37.99 | | 0.05 | 6.62 | 0.98 | | 12.38 | | 14.34 | | 18.74 | | 0.67 | | 0.00 | |  |  |  |
| df | 1 | | 1 | 1 | 1 | | 1 | | 1 | | 1 | | 1 | | 1 | |  |  |  |
| *P-value* | <0.0001 | | 0.82 | 0.01 | 0.32 | | <0.0001 | | <0.0001 | | <0.0001 | | 0.41 | | 0.99 | |  |  |  |
| Stat | Wcl | DPUH | | PPu | PHF | DYPTr | | prepTr | | HDise | | LivDise | | PrLiveM | |  | |  |  |
| *χ^2^* | 1.04 | 0.98 | | 0.78 | 1.82 | 9.03 | | 7.62 | | 8.55 | | 6.77 | | 8.32 | |  | |  |  |
| df | 1 | 1 | | 1 | 1 | 1 | | 1 | | 1 | | 1 | | 1 | |  | |  |  |
| *P-value* | 0.31 | 0.32 | | 0.38 | 0.18 | 0.003 | | 0.006 | | 0.003 | | 0.01 | | 0.004 | |  | |  |  |
| **Notices:** Stat is Statistics, MOL is Mode of living, DHp is do you know how *P.juliflora* introduced, IYH is If yes how was introduced, IIPWI is If *P. juliflora* introduced was intentional, who brought, WIn is Why was *P. juliflora* introduced in your site?, PSreg Preferred site for *P. juliflora* regeneration, BPJ is What benefits you get from *P.juliflora?* IPoNr is which do you think positive or negative impacts of *P.juliflora* higher? DCmU is do communities use *P. juliflora* in your district?, Wcl is Which wealth class mostly uses the *P. juliflora*?, DPUH is does *P. juliflora* use for human food? PPu is Which part of *P. juliflora* use for human food?, PHF is List preparation of human food from *P. juliflora,* DYPTr is Do you think *P. juliflora* is used for traditional medicine? prepTr is the Preparation of *P. juliflora* for traditional medicine?, HDise is a human disease, LivDis is Livestock disease, PrLiveM is a Preparation method for traditional medicine for livestock diseases from *P. juliflora?* | | | | | | | | | | | | | | | | |  |  |  |
