# Supplementary material for: Community perceptions towards invasion of Prosopis juliflora, utilization, and its control options in Afar region, Northeast Ethiopia
Source: PLoS One. 2022 Jan 25;17(1):e0261838. doi: 10.1371/journal.pone.0261838 (PMC8789103; doi:10.1371/journal.pone.0261838)
Supplement: S1 File — (DOCX) [file pone.0261838.s003.docx]

## Appendix 11. Introduction, growth preference phenology, and seed dispersal of *P.juliflora* in South Afar Region

| Variables | | | | | |  | | | | | | |  | |  | | Site | |  | | Response | | |  | |
| --- | --- | --- | --- | --- | --- | --- | --- | --- | --- | --- | --- | --- | --- | --- | --- | --- | --- | --- | --- | --- | --- | --- | --- | --- | --- |
| Earn living modes? | | | | | | District | | | | | | | Dudub | | Kebena | | Kurkura | | Sdihafeghe | | Frequency | | | % | |
| Pastoralist | | | | | | Awash Fentale | | | | | | | 3 | | 21 | | 0 | | 0 | | 24 | | | 15.6 | |
|  | | | | | | Amibara | | | | | | | 0 | | 2 | | 37 | | 37 | | 76 | | | 49.4 | |
| Petty trade | | | | | | Awash Fentale | | | | | | | 0 | | 0 | | 0 | | 0 | | 0 | | | 0.0 | |
|  | | | | | | Amibara | | | | | | | 0 | | 0 | | 2 | | 1 | | 3 | | | 1.9 | |
| Daily laborer | | | | | | Awash Fentale | | | | | | | 1 | | 0 | | 0 | | 0 | | 1 | | | 0.6 | |
|  | | | | | | Amibara | | | | | | | 0 | | 0 | | 9 | | 0 | | 9 | | | 5.8 | |
| Agro-pastoralist | | | | | | Awash Fentale | | | | | | | 18 | | 7 | | 0 | | 0 | | 25 | | | 16.2 | |
|  | | | | | | Amibara | | | | | | | 0 | | 0 | | 7 | | 0 | | 7 | | | 4.5 | |
| Government employee | | | | | | Awash Fentale | | | | | | | 0 | | 1 | | 0 | | 0 | | 1 | | | 0.6 | |
|  | | | | | | Amibara | | | | | | | 0 | | 0 | | 0 | | 0 | | 0 | | | 0.0 | |
| Age of *P.juliflora* in your site? | | | | | |  | | | | | | | | | | | | | | | | | | | |
| 15 | | | | | | Awash Fentale | | | | | 2 | | | | 0 | 0 | | 0 | | 2 | | | 1.3 | | |
|  | | | | | | Amibara | | | | | 0 | | | | 0 | 0 | | 0 | | 0 | | | 0.0 | | |
| 18 | | | | | | Awash Fentale | | | | | 3 | | | | 0 | 0 | | 0 | | 3 | | | 1.9 | | |
|  | | | | | | Amibara | | | | | 0 | | | | 0 | 0 | | 0 | | 0 | | | 0.0 | | |
| 20 | | | | | | Awash Fentale | | | | | 6 | | | | 0 | 0 | | 0 | | 6 | | | 3.9 | | |
|  | | | | | | Amibara | | | | | 0 | | | | 0 | 0 | | 0 | | 0 | | | 0.0 | | |
| 21 | | | | | | Awash Fentale | | | | | 6 | | | | 0 | 0 | | 0 | | 6 | | | 3.9 | | |
|  | | | | | | Amibara | | | | | 0 | | | | 0 | 0 | | 0 | | 0 | | | 0.0 | | |
| 22 | | | | | | Awash Fentale | | | | | 1 | | | | 0 | 0 | | 0 | | 1 | | | 0.6 | | |
|  | | | | | | Amibara | | | | | 0 | | | | 0 | 0 | | 0 | | 0 | | | 0.0 | | |
| 23 | | | | | | Awash Fentale | | | | | 4 | | | | 29 | 0 | | 0 | | 33 | | | 21.4 | | |
|  | | | | | | Amibara | | | | | 0 | | | | 2 | 20 | | 0 | | 22 | | | 14.3 | | |
| 25 | | | | | | Awash Fentale | | | | | 0 | | | | 0 | 0 | | 0 | | 0 | | | 0.0 | | |
|  | | | | | | Amibara | | | | | 0 | | | | 0 | 1 | | 0 | | 1 | | | 0.6 | | |
| 27 | | | | | | Awash Fentale | | | | | 0 | | | | 0 | 0 | | 0 | | 0 | | | 0.0 | | |
|  | | | | | | Amibara | | | | | 0 | | | | 0 | 6 | | 0 | | 6 | | | 3.9 | | |
| 28 | | | | | | Awash Fentale | | | | | 0 | | | | 0 | 0 | | 0 | | 0 | | | 0.0 | | |
|  | | | | | | Amibara | | | | | 0 | | | | 0 | 20 | | 0 | | 20 | | | 13.0 | | |
| 29 | | | | | | Awash Fentale | | | | | 0 | | | | 0 | 0 | | 0 | | 0 | | | 0.0 | | |
|  | | | | | | Amibara | | | | | 0 | | | | 0 | 2 | | 0 | | 2 | | | 1.3 | | |
| 30 | | | | | | Awash Fentale | | | | | 0 | | | | 0 | 0 | | 0 | | 0 | | | 0.0 | | |
|  | | | | | | Amibara | | | | | 0 | | | | 0 | 1 | | 0 | | 1 | | | 0.6 | | |
| 31 | | | | | | Awash Fentale | | | | | 0 | | | | 0 | 0 | | 0 | | 0 | | | 0.0 | | |
|  | | | | | | Amibara | | | | | 0 | | | | 0 | 9 | | 28 | | 37 | | | 24.0 | | |
| 33 | | | | | | Awash Fentale | | | | | 0 | | | | 0 | 0 | | 0 | | 0 | | | 0.0 | | |
|  | | | | | | Amibara | | | | | 0 | | | | 0 | 0 | | 11 | | 11 | | | 7.1 | | |
| 34 | | | | | | Awash Fentale | | | | | 0 | | | | 0 | 0 | | 0 | | 0 | | | 0.0 | | |
|  | | | | | | Amibara | | | | | 0 | | | | 0 | 0 | | 2 | | 2 | | | 1.3 | | |
| Do you know how *P.juliflora* introduced? | | | | | | | | | | | | | | | | | | | | | | | | | |
|  | | | | | |  | | | | |  | | | |  |  | |  | |  | | |  | | |
| Yes | | | | | | Awash Fentale | | | | | 22 | | | | 28 | 0 | | 0 | | 50 | | | 32.7 | | |
|  | | | | | | Amibara | | | | | 0 | | | | 2 | 55 | | 30 | | 87 | | | 56.9 | | |
| No | | | | | | Awash Fentale | | | | | | | | | 1 | 0 | | 0 | | 1 | | | 0.7 | | |
|  | | | | | | Amibara | | | | | | | | | 0 | 4 | | 11 | | 15 | | | 9.8 | | |
| If yes how was introduced? | | | | | | | | | | | | | | | | | | | | | | | | | |
| Local people | | | | | | Awash Fentale | | | | | 0 | | | | 2 | 0 | | 0 | | 2 | | | 1.3 | | |
|  | | | | | | Amibara | | | | | 0 | | | | 0 | 4 | | 6 | | 10 | | | 6.5 | | |
| Natural | | | | | | Awash Fentale | | | | | 0 | | | | 0 | 0 | | 0 | | 0 | | | 0.0 | | |
|  | | | | | | Amibara | | | | | 0 | | | | 0 | 2 | | 3 | | 5 | | | 3.2 | | |
| Foreigners | | | | | | Awash Fentale | | | | | 2 | | | | 3 | 0 | | 0 | | 5 | | | 3.2 | | |
|  | | | | | | Amibara | | | | | 0 | | | | 0 | 11 | | 18 | | 29 | | | 18.8 | | |
| Livestock | | | | | | Awash Fentale | | | | | 20 | | | | 24 | 0 | | 0 | | 44 | | | 28.6 | | |
|  | | | | | | Amibara | | | | | 0 | | | | 2 | 38 | | 6 | | 46 | | | 29.9 | | |
| Wild animals | | | | | | Awash Fentale | | | | | 0 | | | | 0 | 0 | | 0 | | 0 | | | 0.0 | | |
|  | | | | | | Amibara | | | | | 0 | | | | 0 | 1 | | 0 | | 1 | | | 0.6 | | |
| Others | | | | | | Awash Fentale | | | | | 0 | | | | 0 | 0 | | 0 | | 0 | | | 0.0 | | |
|  | | | | | | Amibara | | | | | 0 | | | | 0 | 3 | | 8 | | 11 | | | 7.1 | | |
| If *P.juliflora* introduced was intentional, who brought? | | | | | | | | | | | | | | | | | | | | | | | | | |
| Gov authorities | | | | | | Awash Fentale | | | | | 1 | | | | 9 | 0 | | 0 | | 10 | | | 6.5 | | |
|  | | | | | | Amibara | | | | | 0 | | | | 2 | 13 | | 5 | | 20 | | | 13.0 | | |
| NGO'S | | | | | | Awash Fentale | | | | | 11 | | | | 7 | 0 | | 0 | | 18 | | | 11.7 | | |
|  | | | | | | Amibara | | | | | 0 | | | | 0 | 22 | | 27 | | 49 | | | 31.8 | | |
| Individuals | | | | | | Awash Fentale | | | | | 10 | | | | 13 | 0 | | 0 | | 23 | | | 14.9 | | |
|  | | | | | | Amibara | | | | | 0 | | | | 0 | 24 | | 9 | | 33 | | | 21.4 | | |
|  | | | | | | Awash Fentale | | | | | 22 | | | | 29 | 0 | | 0 | | 51 | | | 33.1 | | |
|  | | | | | | Amibara | | | | | 0 | | | | 2 | 59 | | 41 | | 102 | | | 66.2 | | |
| Why was *P.juliflora* introduced in your site? | | | | | | | | | | | | | | | | | | | | | | | | | |
| Fuel wood | | | | | | Awash Fentale | | | | | 5 | | | | 10 | 0 | | 0 | | 15 | | | 9.7 | | |
|  | | | | | | Amibara | | | | | 0 | | | | 2 | 26 | | 20 | | 48 | | | 31.2 | | |
| Shade | | | | | | Awash Fentale | | | | | 3 | | | | 5 | 0 | | 0 | | 8 | | | 5.2 | | |
|  | | | | | | Amibara | | | | | 0 | | | | 0 | 15 | | 12 | | 27 | | | 17.5 | | |
| Soil and Water Conservation | | | | | | Awash Fentale | | | | | 5 | | | | 9 | 0 | | 0 | | 14 | | | 9.1 | | |
|  | | | | | | Amibara | | | | | 0 | | | | 0 | 2 | | 3 | | 5 | | | 3.2 | | |
| Ornamental | | | | | | Awash Fentale | | | | | 1 | | | |  |  | | 0 | | 1 | | | 0.6 | | |
|  | | | | | | Amibara | | | | | 0 | | | |  |  | | 1 | | 1 | | | 0.6 | | |
| Shelterbelts | | | | | | Awash Fentale | | | | | 1 | | | | 1 | 0 | |  | | 2 | | | 1.3 | | |
|  | | | | | | Amibara | | | | | 0 | | | | 0 | 2 | |  | | 2 | | | 1.3 | | |
| I do not know | | | | | | Awash Fentale | | | | | 7 | | | | 4 | 0 | | 0 | | 11 | | | 7.1 | | |
|  | | | | | | Amibara | | | | | 0 | | | | 0 | 14 | | 5 | | 19 | | | 12.3 | | |
| Topographic preference of *P.juliflora*? | | | | | | | | | | | | | | | | | | | | | | | | | |
| Hilly areas | | | | | | Awash Fentale | | | | | 1 | | | | 0 | 0 | | 0 | | 1 | | | 0.6 | | |
|  | | | | | | Amibara | | | | | 0 | | | | 0 | 0 | | 1 | | 1 | | | 0.6 | | |
| Flat lands | | | | | | Awash Fentale | | | | | 18 | | | | 28 | 0 | | 0 | | 46 | | | 29.9 | | |
|  | | | | | | Amibara | | | | | 0 | | | | 2 | 46 | | 30 | | 78 | | | 50.6 | | |
| Gorges | | | | | | Awash Fentale | | | | | 0 | | | | 0 | 0 | | 0 | | 0 | | | 0.0 | | |
|  | | | | | | Amibara | | | | | 0 | | | | 0 | 0 | | 3 | | 3 | | | 1.9 | | |
| Rivers | | | | | | Amibara | | | | | 3 | | | | 2 | 0 | | 0 | | 5 | | | 3.2 | | |
|  | | | | | | Awash Fentale | | | | | 0 | | | | 0 | 13 | | 7 | | 20 | | | 13.0 | | |
| How do think *P.juliflora* dispersed? | | | | | | | | | | | | | | | | | | | | | | | | | |
| Self-dispersal | | | Awash Fentale | | | | | | | | 1 | | | | 0 | 0 | | 0 | | 1 | | | 0.6 | | |
|  | | | Amibara | | | | | | | | 0 | | | | 0 | 4 | | 6 | | 10 | | | 6.5 | | |
| Livestock | | | Awash Fentale | | | | | | | | 21 | | | | 29 | 0 | | 0 | | 50 | | | 32.5 | | |
|  | | | Amibara | | | | | | | | 0 | | | | 2 | 54 | | 34 | | 90 | | | 58.4 | | |
| Erosion | | | Awash Fentale | | | | | | | | 0 | | | | 1 | 0 | | 0 | | 1 | | | 0.6 | | |
|  | | | Amibara | | | | | | | | 0 | | | | 0 | 0 | | 0 | | 0 | | | 0.0 | | |
| Other | | | Awash Fentale | | | | | | | | 0 | | | | 0 | 0 | | 0 | | 0 | | | 0.0 | | |
|  | | | Amibara | | | | | | | | 0 | | | | 0 | 1 | | 0 | | 1 | | | 0.6 | | |
| Lush growth of *P.juliflora?* | | |  | | | | | | | | | | | | | | | | | | | | | | |
| September | | | Amibara | | | | | | | | 13 | | | | 9 | 0 | | 0 | | 22 | | | 14.3 | | |
|  | | | Awash Fentale | | | | | | | | 0 | | | | 0 | 15 | | 9 | | 24 | | | 15.6 | | |
| October | | | Amibara | | | | | | | | 0 | | | | 0 | 0 | | 0 | | 0 | | | 0.0 | | |
|  | | | Awash Fentale | | | | | | | | 0 | | | | 0 | 0 | | 1 | | 1 | | | 0.6 | | |
|  | | | Amibara | | | | | | | | 0 | | | | 0 | 0 | | 1 | | 1 | | | 0.6 | | |
| November | | | Awash Fentale | | | | | | | | 1 | | | | 0 | 0 | | 0 | | 1 | | | 0.6 | | |
|  | | | Amibara | | | | | | | | 0 | | | | 0 | 0 | | 0 | | 0 | | | 0.0 | | |
| June | | | Awash Fentale | | | | | | | | 0 | | | | 4 | 0 | | 0 | | 4 | | | 2.6 | | |
|  | | | Amibara | | | | | | | | 0 | | | | 0 | 4 | | 0 | | 4 | | | 2.6 | | |
| July | | | Awash Fentale | | | | | | | | 1 | | | | 17 | 0 | | 0 | | 18 | | | 11.7 | | |
|  | | | Amibara | | | | | | | | 0 | | | | 2 | 27 | | 7 | | 36 | | | 23.4 | | |
| August | | | Awash Fentale | | | | | | | | 0 | | | | 0 | 0 | | 0 | | 0 | | | 0.0 | | |
|  | | | Amibara | | | | | | | | 0 | | | | 0 | 7 | | 22 | | 29 | | | 18.8 | | |
| January | | | Awash Fentale | | | | | | | | 0 | | | | 0 | 0 | | 0 | | 0 | | | 0.0 | | |
|  | | | Amibara | | | | | | | | 0 | | | | 0 | 1 | | 0 | | 1 | | | 0.6 | | |
| Februrary | | | Awash Fentale | | | | | | | | 0 | | | | 0 | 0 | | 0 | | 0 | | | 0.0 | | |
|  | | | Amibara | | | | | | | | 0 | | | | 0 | 0 | | 1 | | 1 | | | 0.6 | | |
| December | | | Awash Fentale | | | | | | | | 1 | | | | 0 | 0 | | 0 | | 1 | | | 0.6 | | |
|  | | | Amibara | | | | | | | | 0 | | | | 0 | 0 | | 1 | | 1 | | | 0.6 | | |
| Throughout the year | | | Awash Fentale | | | | | | | | 6 | | | | 0 | 0 | | 0 | | 6 | | | 3.9 | | |
|  | | | Amibara | | | | | | | | 0 | | | | 0 | 5 | | 0 | | 5 | | | 3.2 | | |
| Flower month | | |  | | | | | | | | | | | | | | | | | | | | | | |
| September | | | Awash Fentale | | | | | | | | 0 | | | | 0 | 0 | | 0 | | 0 | | | 0.0 | | |
|  | | | Amibara | | | | | | | | 0 | | | | 0 | 7 | | 12 | | 19 | | | 12.3 | | |
| October | | | Awash Fentale | | | | | | | | 0 | | | | 14 | 0 | | 0 | | 14 | | | 9.1 | | |
|  | | | Amibara | | | | | | | | 0 | | | | 1 | 23 | | 2 | | 26 | | | 16.9 | | |
| November | | | Awash Fentale | | | | | | | | 1 | | | | 0 | 0 | | 0 | | 1 | | | 0.6 | | |
|  | | | Amibara | | | | | | | | 0 | | | | 0 | 2 | | 2 | | 4 | | | 2.6 | | |
| May | | | Awash Fentale | | | | | | | | 1 | | | | 0 | 0 | | 0 | | 1 | | | 0.6 | | |
|  | | | Amibara | | | | | | | | 0 | | | | 1 | 2 | | 0 | | 3 | | | 1.9 | | |
| April | | | Awash Fentale | | | | | | | | 0 | | | | 3 | 0 | | 0 | | 3 | | | 1.9 | | |
|  | | | Amibara | | | | | | | | 0 | | | | 0 | 0 | | 3 | | 3 | | | 1.9 | | |
| June | | | Awash Fentale | | | | | | | | 0 | | | | 3 | 0 | | 0 | | 3 | | | 1.9 | | |
|  | | | Amibara | | | | | | | | 0 | | | | 0 | 1 | | 0 | | 1 | | | 0.6 | | |
| July | | | Awash Fentale | | | | | | | | 1 | | | | 2 | 0 | | 0 | | 3 | | | 1.9 | | |
|  | | | Amibara | | | | | | | | 0 | | | | 0 | 5 | | 4 | | 9 | | | 5.8 | | |
| August | | | Awash Fentale | | | | | | | | 0 | | | | 0 | 0 | | 0 | | 0 | | | 0.0 | | |
|  | | | Amibara | | | | | | | | 0 | | | | 0 | 0 | | 12 | | 12 | | | 7.8 | | |
| March | | | Awash Fentale | | | | | | | | 0 | | | | 0 | 0 | | 0 | | 0 | | | 0.0 | | |
|  | | | Amibara | | | | | | | | 0 | | | | 0 | 5 | | 1 | | 6 | | | 3.9 | | |
| January | | | Awash Fentale | | | | | | | | 0 | | | | 0 | 0 | | 0 | | 0 | | | 0.0 | | |
|  | | | Amibara | | | | | | | | 0 | | | | 0 | 1 | | 1 | | 2 | | | 1.3 | | |
| February | | | Awash Fentale | | | | | | | | 1 | | | | 0 | 0 | | 0 | | 1 | | | 0.6 | | |
|  | | | Amibara | | | | | | | | 0 | | | | 0 | 0 | | 2 | | 2 | | | 1.3 | | |
| December | | | Awash Fentale | | | | | | | | 11 | | | | 8 | 0 | | 0 | | 19 | | | 12.3 | | |
|  | | | Amibara | | | | | | | | 0 | | | | 0 | 13 | | 2 | | 15 | | | 9.7 | | |
| Throughout the year | | | Awash Fentale | | | | | | | | 7 | | | | 0 | 0 | | 0 | | 7 | | | 4.5 | | |
|  | | | Amibara | | | | | | | | 0 | | | | 0 | 0 | | 0 | | 0 | | | 0.0 | | |
| Seed set of *P.juliflora*? | | |  | | | | | | | | | | | | | | | | | | | | | | |
| September | | | Awash Fentale | | | | | | | | 1 | | | | 0 | 0 | | 0 | | 1 | | | 0.6 | | |
|  | | | Amibara | | | | | | | | 0 | | | | 0 | 5 | | 5 | | 10 | | | 6.5 | | |
| October | | | Awash Fentale | | | | | | | | 0 | | | | 0 | 0 | | 0 | | 0 | | | 0.0 | | |
|  | | | Amibara | | | | | | | | 0 | | | | 0 | 5 | | 2 | | 7 | | | 4.5 | | |
| November | | | Awash Fentale | | | | | | | | 1 | | | | 0 | 0 | | 0 | | 1 | | | 0.6 | | |
|  | | | Amibara | | | | | | | | 0 | | | | 0 | 2 | | 3 | | 5 | | | 3.2 | | |
| May | | | Awash Fentale | | | | | | | | 1 | | | | 0 | 0 | | 0 | | 1 | | | 0.6 | | |
|  | | | Amibara | | | | | | | | 0 | | | | 0 | 2 | | 10 | | 12 | | | 7.8 | | |
| April | | | Awash Fentale | | | | | | | | 0 | | | | 17 | 0 | | 0 | | 17 | | | 11.0 | | |
|  | | | Amibara | | | | | | | | 0 | | | | 2 | 23 | | 1 | | 26 | | | 16.9 | | |
| June | | | Awash Fentale | | | | | | | | 0 | | | | 2 | 0 | | 0 | | 2 | | | 1.3 | | |
|  | | | Amibara | | | | | | | | 0 | | | | 0 | 2 | | 1 | | 3 | | | 1.9 | | |
| July | | | Awash Fentale | | | | | | | | 0 | | | | 2 | 0 | | 0 | | 2 | | | 1.3 | | |
|  | | | Amibara | | | | | | | | 0 | | | | 0 | 3 | | 4 | | 7 | | | 4.5 | | |
| August | | | Awash Fentale | | | | | | | | 0 | | | | 1 | 0 | | 0 | | 1 | | | 0.6 | | |
|  | | | Amibara | | | | | | | | 0 | | | | 0 | 0 | | 9 | | 9 | | | 5.8 | | |
| March | | | Awash Fentale | | | | | | | | 0 | | | | 0 | 0 | | 0 | | 0 | | | 0.0 | | |
|  | | | Amibara | | | | | | | | 0 | | | | 0 | 1 | | 1 | | 2 | | | 1.3 | | |
| January | | | Awash Fentale | | | | | | | | 0 | | | | 0 | 0 | | 0 | | 0 | | | 0.0 | | |
|  | | | Amibara | | | | | | | | 0 | | | | 0 | 2 | | 1 | | 3 | | | 1.9 | | |
| February | | | Awash Fentale | | | | | | | | 13 | | | | 8 | 0 | | 0 | | 21 | | | 13.6 | | |
|  | | | Amibara | | | | | | | | 0 | | | | 0 | 13 | | 2 | | 15 | | | 9.7 | | |
| December | | | Awash Fentale | | | | | | | | 0 | | | | 0 | 0 | | 0 | | 0 | | | 0.0 | | |
|  | | | Amibara | | | | | | | | 0 | | | | 0 | 1 | | 2 | | 3 | | | 1.9 | | |
| Throughout the year | | | Awash Fentale | | | | | | | | 6 | | | | 0 | 0 | | 0 | | 6 | | | 3.9 | | |
|  | | | Amibara | | | | | | | | 0 | | | | 0 | 0 | | 0 | | 0 | | | 0.0 | | |
| Preferred site for *P.juliflora* regeneration (establishment)? | | | | | | | | | | | | | | | | | | | | | | | | | |
| Homestead | | | Awash Fentale | | | | | | | | 3 | | | | 3 | 0 | | 0 | | 6 | | | 3.9 | | |
|  | | | Amibara | | | | | | | | 0 | | | | 0 | 2 | | 19 | | 21 | | | 13.6 | | |
| Road sides | | | Awash Fentale | | | | | | | | 3 | | | | 6 | 0 | | 0 | | 9 | | | 5.8 | | |
|  | | | Amibara | | | | | | | | 0 | | | | 1 | 6 | | 6 | | 13 | | | 8.4 | | |
| Wetlands | | | Awash Fentale | | | | | | | | 7 | | | | 2 | 0 | | 0 | | 9 | | | 5.8 | | |
|  | | | Amibara | | | | | | | | 0 | | | | 0 | 2 | | 1 | | 3 | | | 1.9 | | |
| Range lands | | | Awash Fentale | | | | | | | | 6 | | | | 17 | 0 | | 0 | | 23 | | | 14.9 | | |
|  | | | Amibara | | | | | | | | 0 | | | | 1 | 28 | | 1 | | 30 | | | 19.5 | | |
| Around crop lands | | | Awash Fentale | | | | | | | | 3 | | | | 2 | 0 | | 0 | | 5 | | | 3.2 | | |
|  | | | Amibara | | | | | | | | 0 | | | | 0 | 7 | | 0 | | 7 | | | 4.5 | | |
| Along rivers | | | Awash Fentale | | | | | | | | 0 | | | | 0 | 0 | | 0 | | 0 | | | 0.0 | | |
|  | | | Amibara | | | | | | | | 0 | | | | 0 | 13 | | 14 | | 27 | | | 17.5 | | |
| Urban areas | | | Awash Fentale | | | | | | | | 0 | | | | 0 | 0 | | 0 | | 0 | | | 0.0 | | |
| Livestock most dispersed *P.juliflora* seeds? | | | | | | | | | | | | | | | | | | | | | | | | | |
| Cattle | | | Awash Fentale | | | | | | | | 14 | | | | 23 | 0 | | 0 | | 37 | | | 24.0 | | |
|  | | | Amibara | | | | | | | | 0 | | | | 2 | 29 | | 12 | | 43 | | | 27.9 | | |
| Camels | | | Awash Fentale | | | | | | | | 0 | | | | 0 | 0 | | 0 | | 0 | | | 0.0 | | |
|  | | | Amibara | | | | | | | | 0 | | | | 0 | 6 | | 0 | | 6 | | | 3.9 | | |
| Goats | | | Awash Fentale | | | | | | | | 7 | | | | 3 | 0 | | 0 | | 10 | | | 6.5 | | |
|  | | | Amibara | | | | | | | | 0 | | | | 0 | 21 | | 24 | | 45 | | | 29.2 | | |
| Sheep | | | Awash Fentale | | | | | | | | 1 | | | | 3 | 0 | | 0 | | 4 | | | 2.6 | | |
|  | | | Amibara | | | | | | | | 0 | | | | 0 | 3 | | 5 | | 8 | | | 5.2 | | |
| Wild animal most dispersed *P.juliflora?* | | | | | | | | | | | | | | | | | | | | | | | | | |
| Warthog | | | Awash Fentale | | | | | | | | 11 | | | | 25 | 0 | | 0 | | 36 | | | 23.4 | | |
|  | | | Amibara | | | | | | | | 0 | | | | 2 | 34 | | 35 | | 71 | | | 46.1 | | |
| Gazelle | | | Awash Fentale | | | | | | | | 1 | | | | 0 | 0 | | 0 | | 1 | | | 0.6 | | |
|  | | | Amibara | | | | | | | | 0 | | | | 0 | 0 | | 1 | | 1 | | | 0.6 | | |
| Kudus | | | Awash Fentale | | | | | | | | 0 | | | | 1 | 0 | | 0 | | 1 | | | 0.6 | | |
|  | | | Amibara | | | | | | | | 0 | | | | 0 | 1 | | 0 | | 1 | | | 0.6 | | |
| Monkeys | | | Awash Fentale | | | | | | | | 10 | | | | 3 | 0 | | 0 | | 13 | | | 8.4 | | |
|  | | | Amibara | | | | | | | | 0 | | | | 0 | 23 | | 4 | | 27 | | | 17.5 | | |
| Dikdik | | | Awash Fentale | | | | | | | | 0 | | | | 0 | 0 | | 0 | | 0 | | | 0.0 | | |
|  | | | Amibara | | | | | | | | 0 | | | | 0 | 1 | | 0 | | 1 | | | 0.6 | | |
| Birds | | | Awash Fentale | | | | | | | | 0 | | | | 0 | 0 | | 0 | | 0 | | | 0.0 | | |
|  | | | Amibara | | | | | | | | 0 | | | | 0 | 0 | | 1 | | 1 | | | 0.6 | | |
| Which dispersal agents responsible for *P.juliflora* dispersal? | | | | | | | | | | | | | | | | | | | | | | | | | |
| Floods | | | Amibara | | | | | | | | 21 | | | | 29 | 0 | | 0 | | 50 | | | 32.5 | | |
|  | | | Awash Fentale | | | | | | | | 0 | | | | 2 | 57 | | 40 | | 99 | | | 64.3 | | |
| Winds | | | Amibara | | | | | | | | 1 | | | | 0 | 0 | | 0 | | 1 | | | 0.6 | | |
|  | | | Awash Fentale | | | | | | | | 0 | | | | 0 | 2 | | 0 | | 2 | | | 1.3 | | |
| Other | | | Amibara | | | | | | | | 0 | | | | 0 | 0 | | 0 | | 0 | | | 0.0 | | |
|  | | | Awash Fentale | | | | | | | | 0 | | | | 0 | 0 | | 1 | | 1 | | | 0.6 | | |
| Month of maximum seed set/dispersal for *P.juliflora?* | | | | | | | | | | | | | | | | | | | | | | | | | |
| September | | | Awash Fentale | | | | | | | | 1 | | | | 0 | 0 | | 0 | | 1 | | | 0.6 | | |
|  | | | Amibara | | | | | | | | 0 | | | | 0 | 7 | | 5 | | 12 | | | 7.8 | | |
| October | | | Awash Fentale | | | | | | | | 3 | | | | 0 | 0 | | 0 | | 3 | | | 1.9 | | |
|  | | | Amibara | | | | | | | | 0 | | | | 0 | 2 | | 1 | | 3 | | | 1.9 | | |
| November | | | Awash Fentale | | | | | | | | 3 | | | | 0 | 0 | | 0 | | 3 | | | 1.9 | | |
|  | | | Amibara | | | | | | | | 0 | | | | 0 | 2 | | 3 | | 5 | | | 3.2 | | |
| May | | | Awash Fentale | | | | | | | | 3 | | | | 9 | 0 | | 0 | | 12 | | | 7.8 | | |
|  | | | Amibara | | | | | | | | 0 | | | | 0 | 20 | | 23 | | 43 | | | 27.9 | | |
| June | | | Awash Fentale | | | | | | | | 2 | | | | 1 | 0 | | 0 | | 3 | | | 1.9 | | |
|  | | | Amibara | | | | | | | | 0 | | | | 0 | 6 | | 1 | | 7 | | | 4.5 | | |
| August | | | Awash Fentale | | | | | | | | 0 | | | | 0 | 0 | | 0 | | 0 | | | 0.0 | | |
|  | | | Amibara | | | | | | | | 0 | | | | 0 | 10 | | 5 | | 15 | | | 9.7 | | |
| March | | | Awash Fentale | | | | | | | | 0 | | | | 1 | 0 | | 0 | | 1 | | | 0.6 | | |
|  | | | Amibaraq | | | | | | | | 0 | | | | 0 | 1 | | 0 | | 1 | | | 0.6 | | |
| February | | | Awash Fentale | | | | | | | | 6 | | | | 17 | 0 | | 0 | | 23 | | | 14.9 | | |
|  | | | Amibara | | | | | | | | 0 | | | | 2 | 11 | | 1 | | 14 | | | 9.1 | | |
| December | | | Awash Fentale | | | | | | | | 2 | | | | 1 | 0 | | 0 | | 3 | | | 1.9 | | |
|  | | | Amibara | | | | | | | | 0 | | | | 0 | 0 | | 2 | | 2 | | | 1.3 | | |
| Throughout the year | | | Awash Fentale | | | | | | | | 2 | | | | 0 | 0 | | 0 | | 2 | | | 1.3 | | |
|  | | | Amibara | | | | | | | | 0 | | | | 0 | 0 | | 0 | | 0 | | | 0.0 | | |
| Major *P.juliflora* regeneration? | | |  | | | | | | | | | | | | | | | | | | | | | | |
| Seeds | | | Awash Fentale | | | | | | | | 19 | | | | 11 | 0 | | 0 | | 30 | | | 19.5 | | |
|  | | | Amibara | | | | | | | | 0 | | | | 0 | 37 | | 19 | | 56 | | | 36.4 | | |
| Coppicing from cut stems | | | Awash Fentale | | | | | | | | 3 | | | | 18 | 0 | | 0 | | 21 | | | 13.6 | | |
|  | | | Amibara | | | | | | | | 0 | | | | 2 | 20 | | 22 | | 44 | | | 28.6 | | |
| Regeneration from roots | | | Awash Fentale | | | | | | | | 0 | | | | 0 | 0 | | 0 | | 0 | | | 0.0 | | |
|  | | | Amibara | | | | | | | | 0 | | | | 0 | 2 | | 0 | | 2 | | | 1.3 | | |
| Did you count *P.juliflora* coppices? | | | | | | | | | | | | | | | | | | | | | | | | | |
| Yes | | | Awash Fentale | | | | | | | | 8 | | | | 14 | 0 | | 0 | | 22 | | | 14.3 | | |
|  | | | Amibara | | | | | | | | 0 | | | | 0 | 6 | | 2 | | 8 | | | 5.2 | | |
| No | | | Awash Fentale | | | | | | | | 14 | | | | 15 | 0 | | 0 | | 29 | | | 18.8 | | |
|  | | | Amibara | | | | | | | | 0 | | | | 2 | 53 | | 39 | | 94 | | | 61.0 | | |
| If yes number of coppices? | | | | | | | | | | |  | | | |  |  | |  | |  | | |  | | |
| I do not know | | | Awash Fentale | | | | | | | | 16 | | | | 28 | 0 | | 0 | | 44 | | | 28.6 | | |
|  | | | Amibara | | | | | | | | 0 | | | | 2 | 49 | | 40 | | 91 | | | 59.1 | | |
| 1 | | | Awash Fentale | | | | | | | | 0 | | | | 1 | 0 | | 0 | | 1 | | | 0.6 | | |
|  | | | Amibara | | | | | | | | 0 | | | | 0 | 0 | | 0 | | 0 | | | 0.0 | | |
| 4 | | | Awash Fentale | | | | | | | | 0 | | | | 0 | 0 | | 0 | | 0 | | | 0.0 | | |
|  | | | Amibara | | | | | | | | 0 | | | | 0 | 7 | | 1 | | 8 | | | 5.2 | | |
| 5 | | | Awash Fentale | | | | | | | | 2 | | | | 1 | 0 | | 0 | | 3 | | | 1.9 | | |
|  | | | Amibara | | | | | | | | 0 | | | | 0 | 2 | | 0 | | 2 | | | 1.3 | | |
| 6 | | | Awash Fentale | | | | | | | | 2 | | | | 0 | 0 | | 0 | | 2 | | | 1.3 | | |
|  | | | Amibara | | | | | | | | 0 | | | | 0 | 0 | | 0 | | 0 | | | 0.0 | | |
| 7 | | | Awash Fentale | | | | | | | | 1 | | | | 0 | 0 | | 0 | | 1 | | | 0.6 | | |
|  | | | Amibara | | | | | | | | 0 | | | | 0 | 0 | | 0 | | 0 | | | 0.0 | | |
| 20 | | | Awash Fentale | | | | | | | | 1 | | | | 0 | 0 | | 0 | | 1 | | | 0.6 | | |
|  | | | Amibara | | | | | | | | 0 | | | | 0 | 0 | | 0 | | 0 | | | 0.0 | | |
| 25 | | | Awash Fentale | | | | | | | | 0 | | | | 0 | 0 | | 0 | | 0 | | | 0.0 | | |
|  | | | Amibara | | | | | | | | 0 | | | | 0 | 1 | | 0 | | 1 | | | 0.6 | | |
| Appendix 12. Ecosystem services, use and impacts *of P.juliflora* in South Afar Region | | | | | | | | | | | | | | | | | | | | | | | | | |
| What benefits you get from *P.juliflora?* | | | | | | | | | | | | | | |  |  | |  | |  | | | 0.0 | | |
| Shade livestock | | | | | Awash Fentale | | | | | | 4 | | | | 5 | 0 | | 0 | | 9 | | | 5.8 | | |
|  | | | | | Amibara | | | | | | 0 | | | | 2 | 3 | | 6 | | 11 | | | 7.1 | | |
| Shade human beings | | | | | Awash Fentale | | | | | | 1 | | | | 1 | 0 | | 0 | | 2 | | | 1.3 | | |
|  | | | | | Amibara | | | | | | 0 | | | | 0 | 4 | | 0 | | 4 | | | 2.6 | | |
| Fuel wood | | | | | Awash Fentale | | | | | | 6 | | | | 13 | 0 | | 0 | | 19 | | | 12.3 | | |
|  | | | | | Amibara | | | | | | 0 | | | | 0 | 45 | | 30 | | 75 | | | 48.7 | | |
| Furniture | | | | | Awash Fentale | | | | | | 0 | | | | 1 | 0 | | 0 | | 1 | | | 0.6 | | |
|  | | | | | Amibara | | | | | | 0 | | | | 0 | 0 | | 0 | | 0 | | | 0.0 | | |
| House construction | | | | | Awash Fentale | | | | | | 3 | | | | 0 | 0 | | 0 | | 3 | | | 1.9 | | |
|  | | | | | Amibara | | | | | | 0 | | | | 0 | 6 | | 0 | | 6 | | | 3.9 | | |
| Live Fencing | | | | | Awash Fentale | | | | | | 3 | | | | 7 | 0 | | 0 | | 10 | | | 6.5 | | |
|  | | | | | Amibara | | | | | | 0 | | | | 0 | 0 | | 2 | | 2 | | | 1.3 | | |
| Soil and Water Conservation | | | | | Awash Fentale | | | | | | 1 | | | | 0 | 0 | | 0 | | 1 | | | 0.6 | | |
|  | | | | | Amibara | | | | | | 0 | | | | 0 | 0 | | 1 | | 1 | | | 0.6 | | |
| Ameliorating effects | | | | | Awash Fentale | | | | | | 0 | | | | 0 | 0 | | 0 | | 0 | | | 0.0 | | |
|  | | | | | Amibara | | | | | | 0 | | | | 0 | 1 | | 0 | | 1 | | | 0.6 | | |
| Shelterbelt | | | | | Awash Fentale | | | | | | 0 | | | | 0 | 0 | | 0 | | 0 | | | 0.0 | | |
|  | | | | | Amibara | | | | | | 0 | | | | 0 | 0 | | 1 | | 1 | | | 0.6 | | |
| Fodder | | | | | Awash Fentale | | | | | | 2 | | | | 0 | 0 | | 0 | | 2 | | | 1.3 | | |
|  | | | | | Amibara | | | | | | 0 | | | | 0 | 0 | | 0 | | 0 | | | 0.0 | | |
| Combating desertification | | | | | Awash Fentale | | | | | | 2 | | | | 3 | 0 | | 0 | | 5 | | | 3.2 | | |
|  | | | | | Amibara | | | | | | 0 | | | | 0 | 0 | | 1 | | 1 | | | 0.6 | | |
| Which do you think positive or negative impacts of *P.juliflora* higher? | | | | | | | | | | | | | | | |  | |  | |  | | |  | | |
| Positive | | | | Awash Fentale | | | | | | | 2 | | | | 1 | 0 | | 0 | | 3 | | | 1.9 | | |
|  | | | | Amibara | | | | | | | 0 | | | | 0 | 1 | | 0 | | 1 | | | 0.6 | | |
| Negative | | | | Awash Fentale | | | | | | | 20 | | | | 29 | 0 | | 0 | | 49 | | | 31.8 | | |
|  | | | | Amibara | | | | | | | 0 | | | | 2 | 57 | | 40 | | 99 | | | 64.3 | | |
| I do not know | | | | Awash Fentale | | | | | | | 0 | | | | 0 | 0 | | 0 | | 0 | | | 0.0 | | |
|  | | | | Amibara | | | | | | | 0 | | | | 0 | 1 | | 1 | | 2 | | | 1.3 | | |
| Do communities use *P.juliflora* in your district? | | | | | | | | | | | | | | |  |  | |  | |  | | | 0.0 | | |
| Yes | | | | | Awash Fentale | | | | | | 22 | | | | 30 | 0 | | 0 | | 52 | | | 33.8 | | |
|  | | | | | Amibara | | | | | | 0 | | | | 2 | 57 | | 40 | | 99 | | | 64.3 | | |
| No | | | | | Awash Fentale | | | | | | 0 | | | | 0 | 0 | | 0 | | 0 | | | 0.0 | | |
|  | | | | | Amibara | | | | | | 0 | | | | 0 | 2 | | 1 | | 3 | | | 1.9 | | |
| Which wealth class mostly uses the *P.juliflora*? | | | | | | | | | | | | | | |  |  | |  | |  | | | 0.0 | | |
| Poor | | | | | Awash Fentale | | | | | | 16 | | | | 25 | 0 | | 0 | | 41 | | | 26.6 | | |
|  | | | | | Amibara | | | | | | 0 | | | | 2 | 53 | | 31 | | 86 | | | 55.8 | | |
| Rich | | | | | Awash Fentale | | | | | | 3 | | | | 3 | 0 | | 0 | | 6 | | | 3.9 | | |
|  | | | | | Amibara | | | | | | 0 | | | | 0 | 3 | | 0 | | 3 | | | 1.9 | | |
| Medium | | | | | Awash Fentale | | | | | | 3 | | | | 2 | 0 | | 0 | | 5 | | | 3.2 | | |
|  | | | | | Amibara | | | | | | 0 | | | | 0 | 3 | | 10 | | 13 | | | 8.4 | | |
| Does *P.juliflora* use for human food? | | | | | | | | | | |  | | | |  |  | |  | |  | | |  | | |
| Yes | | | | | | Awash Fentale | | | | | 1 | | | | 13 | 0 | | 0 | | 14 | | | 9.1 | | |
|  | | | | | | Amibara | | | | | 0 | | | | 2 | 18 | | 3 | | 23 | | | 14.9 | | |
| No | | | | | | Awash Fentale | | | | | 21 | | | | 17 | 0 | | 0 | | 38 | | | 24.7 | | |
|  | | | | | | Amibara | | | | | 0 | | | | 0 | 41 | | 38 | | 79 | | | 51.3 | | |
| Which part of *P.juliflora* use for human food? | | | | | | | | | | | | | | |  |  | |  | |  | | |  | | |
| Leaf | | | | | | Awash Fentale | | | | | 1 | | | | 0 | 0 | | 0 | | 1 | | | 0.6 | | |
|  | | | | | | Amibara | | | | | 0 | | | | 0 | 0 | | 4 | | 4 | | | 2.6 | | |
| Seeds | | | | | | Awash Fentale | | | | | 21 | | | | 30 | 0 | | 0 | | 51 | | | 33.1 | | |
|  | | | | | | Amibara | | | | | 0 | | | | 2 | 59 | | 37 | | 98 | | | 63.6 | | |
| List preparation of human food from *P.juliflora* | | | | | | | | | | | | | | | | | | | | | | | | | |
| Pounding | | | | | | Awash Fentale | | | | | 1 | | | | 1 | 0 | | 0 | | 2 | | | 1.3 | | |
|  | | | | | | Amibara | | | | | 0 | | | | 2 | 6 | | 3 | | 11 | | | 7.1 | | |
| Squeezing | | | | | | Awash Fentale | | | | | 0 | | | | 7 | 0 | | 0 | | 7 | | | 4.5 | | |
|  | | | | | | Amibara | | | | | 0 | | | | 0 | 0 | | 0 | | 0 | | | 0.0 | | |
| Not used | | | | | | Awash Fentale | | | | | 21 | | | | 22 | 0 | | 0 | | 43 | | | 27.9 | | |
|  | | | | | | Amibara | | | | | 0 | | | | 0 | 53 | | 38 | | 91 | | | 59.1 | | |
| Do you think *P.juliflora* for traditional medicine? | | | | | | | | | | | | | | |  |  | |  | |  | | |  | | |
| Yes | | | | | | Awash Fentale | | | | | 7 | | | | 7 | 0 | | 0 | | 14 | | | 9.1 | | |
|  | | | | | | Amibara | | | | | 0 | | | | 0 | 3 | | 11 | | 14 | | | 9.1 | | |
| No | | | | | | Awash Fentale | | | | | 12 | | | | 22 | 0 | | 0 | | 34 | | | 22.1 | | |
|  | | | | | | Amibara | | | | | 0 | | | | 2 | 54 | | 29 | | 85 | | | 55.2 | | |
| I do not know | | | | | | Awash Fentale | | | | | 3 | | | | 1 | 0 | | 0 | | 4 | | | 2.6 | | |
|  | | | | | | Amibara | | | | | 0 | | | | 0 | 2 | | 1 | | 3 | | | 1.9 | | |
| Which part of *P.juliflora* used for traditional medicine? | | | | | | | | | | | | | | | |  | |  | |  | | |  | | |
| seeds/pods | | | | Awash Fentale | | | | | | | 8 | | | | 6 | 0 | | 0 | | 14 | | | 9.1 | | |
|  | | | | Amibara | | | | | | | 0 | | | | 0 | 3 | | 11 | | 14 | | | 9.1 | | |
| Leaf | | | | Awash Fentale | | | | | | | 14 | | | | 24 | 0 | | 0 | | 38 | | | 24.7 | | |
|  | | | | Amibara | | | | | | | 0 | | | | 2 | 56 | | 30 | | 88 | | | 57.1 | | |
| Preparation of *P.julifolra* for traditional medicine for human cure? | | | | | | | | | | | | | | | | | | | | | | | | | |
| Pounding | | Awash Fentale | | | | | | | | | 8 | | | | 5 | 0 | | 0 | | 13 | | | 8.4 | | |
|  | | Amibara | | | | | | | | | 0 | | | | 0 | 2 | | 9 | | 11 | | | 7.1 | | |
| I do not know | | Awash Fentale | | | | | | | | | 14 | | | | 25 | 0 | | 0 | | 39 | | | 25.3 | | |
|  | | Amibara | | | | | | | | | 0 | | | | 2 | 57 | | 32 | | 91 | | | 59.1 | | |
| Human diseases cured by traditional medicine of *P.juliflora?* | | | | | | | | | | | | | | | | | | | | | | | | | |
| Wound | | Awash Fentale | | | | | | | | | 8 | | | | 5 | 0 | | 0 | | 13 | | | 8.4 | | |
|  | | Amibara | | | | | | | | | 0 | | | | 0 | 2 | | 6 | | 8 | | | 5.2 | | |
| I do not know | | Awash Fentale | | | | | | | | | 14 | | | | 25 | 0 | | 0 | | 39 | | | 25.3 | | |
|  | | Amibara | | | | | | | | | 0 | | | | 2 | 57 | | 34 | | 93 | | | 60.4 | | |
| Livestock diseases cured by traditional medicine of *P.juliflora?* | | | | | | | | | | | | | | | |  | |  | |  | | | 0.0 | | |
| Wound | | Awash Fentale | | | | | | | | | 6 | | | | 6 | 0 | | 0 | | 12 | | | 7.8 | | |
|  | | Amibara | | | | | | | | | 0 | | | | 0 | 0 | | 6 | | 6 | | | 3.9 | | |
| I do not know | | Awash Fentale | | | | | | | | | 16 | | | | 24 | 0 | | 0 | | 40 | | | 26.0 | | |
|  | | Amibara | | | | | | | | | 0 | | | | 2 | 59 | | 34 | | 95 | | | 61.7 | | |
| Preparation method for traditional medicine for livestock diseases from *P.juliflora?* | | | | | | | | | | | | | | | | | | | | | | | | | |
| Pounding | | Awash Fentale | | | | | | | | | 6 | | | | 6 | 0 | | 0 | | 12 | | | 7.8 | | |
|  | | Amibara | | | | | | | | | 0 | | | | 0 | 0 | | 7 | | 7 | | | 4.5 | | |
| I do not know | | Awash Fentale | | | | | | | | | 16 | | | | 24 | 0 | | 0 | | 40 | | | 26.0 | | |
|  | | Amibara | | | | | | | | | 0 | | | | 2 | 59 | | 33 | | 94 | | | 61.0 | | |
| What are impacts of *P.juliflora?* | | | | | | | | | | | | | | |  |  | |  | |  | | | 0.0 | | |
| Woody weedy in agricultural lands | | | | | | | Awash Fentale | | | | | 7 | | | 5 | 0 | | 0 | | 12 | | | 7.8 | | |
|  | | | | | | | Amibara | | | | | 0 | | | 0 | 3 | | 4 | | 7 | | | 4.5 | | |
| Encroachments into grazing lands | | | | | | | Awash Fentale | | | | | 10 | | | 20 | 0 | | 0 | | 30 | | | 19.5 | | |
|  | | | | | | | Amibara | | | | | 0 | | | 2 | 40 | | 21 | | 63 | | | 40.9 | | |
| Blocking roads of livestock | | | | | | | Awash Fentale | | | | | 0 | | | 2 | 0 | | 0 | | 2 | | | 1.3 | | |
|  | | | | | | | Amibara | | | | | 0 | | | 0 | 12 | | 10 | | 22 | | | 14.3 | | |
| Blocking roads of human beings | | | | | | | Awash Fentale | | | | | 0 | | | 2 | 0 | | 0 | | 2 | | | 1.3 | | |
|  | | | | | | | Amibara | | | | | 0 | | | 0 | 4 | | 5 | | 9 | | | 5.8 | | |
| Invasion of water courses and drying up rivers and water tables | | | | | | | Awash Fentale | | | | | 0 | | | 0 | 0 | | 0 | | 0 | | | 0.0 | | |
|  | | | | | | | Amibara | | | | | 0 | | | 1 | 0 | | 0 | | 1 | | | 0.6 | | |
| Lack of aesthetic value due to its monoculture | | | | | | | Awash Fentale | | | | | 2 | | | 0 | 0 | | 0 | | 2 | | | 1.3 | | |
|  | | | | | | | Amibara | | | | | 0 | | | 0 | 0 | | 0 | | 0 | | | 0.0 | | |
|  | | | | | | | Awash Fentale | | | | | 3 | | | 0 | 0 | | 0 | | 3 | | | 1.9 | | |
|  | | | | | | | Amibara | | | | | 0 | | | 0 | 0 | | 0 | | 0 | | | 0.0 | | |
| Negative effects of *P.juliflora* on livestock? | | | | | | | | | | | | | | |  |  | |  | |  | | |  | | |
| Animal get injured | | | | | Awash Fentale | | | | | | 9 | | | | 14 | 0 | | 0 | | 23 | | | 15 | | |
|  | | | | | Amibara | | | | | | 0 | | | | 0 | 13 | | 28 | | 41 | | | 27 | | |
| Animal die from eating | | | | | Awash Fentale | | | | | | 8 | | | | 1 | 0 | | 0 | | 9 | | | 6 | | |
|  | | | | | Amibara | | | | | | 0 | | | | 0 | 17 | | 5 | | 22 | | | 14 | | |
| Animals get diarrhea | | | | | Awash Fentale | | | | | | 0 | | | | 5 | 0 | | 0 | | 5 | | | 3 | | |
|  | | | | | Amibara | | | | | | 0 | | | | 2 | 7 | | 2 | | 11 | | | 7 | | |
| Animals get paralyzed | | | | | Awash Fentale | | | | | | 0 | | | | 0 | 0 | | 0 | | 0 | | | 0 | | |
|  | | | | | Amibara | | | | | | 0 | | | | 0 | 2 | | 2 | | 4 | | | 3 | | |
| It is poisoning/toxic effects | | | | | Awash Fentale | | | | | | 5 | | | | 10 | 0 | | 0 | | 15 | | | 9.7 | | |
|  | | | | | Amibara | | | | | | 0 | | | | 0 | 20 | | 3 | | 23 | | | 14.9 | | |
| Negative effects of *P.juliflora* on plants? | | | | | | | | | | | | | | |  |  | |  | |  | | |  | | |
| Reduce of plant diversity | | | | | | | | | | 1 | 4 | | | | 25 | 0 | | 0 | | 29 | | | 18.8 | | |
|  | | | | | | | | | | 2 | 0 | | | | 2 | 31 | | 26 | | 59 | | | 38.3 | | |
| Toxic effects on plant species | | | | | | | | | | 1 | 15 | | | | 5 | 0 | | 0 | | 20 | | | 13.0 | | |
|  | | | | | | | | | | 2 | 0 | | | | 0 | 25 | | 8 | | 33 | | | 21.4 | | |
| Overtake the site and inhibit other plant species not to grow | | | | | | | | | | 1 | 2 | | | | 0 | 0 | | 0 | | 2 | | | 1.3 | | |
|  | | | | | | | | | | 2 | 0 | | | | 0 | 2 | | 6 | | 8 | | | 5.2 | | |
| Others | | | | | | | | | | 1 | 1 | | | | 0 | 0 | | 0 | | 1 | | | 0.6 | | |
|  | | | | | | | | | | 2 | 0 | | | | 0 | 1 | | 0 | | 1 | | | 0.6 | | |
| What impacts does on *P.juliflora* on plants? | | | | | | | | | | | | | | |  |  | |  | |  | | |  | | |
| Positive | | | | | | Awash Fentale | | | | | 9 | | | | 23 | 0 | | 0 | | 32 | | | 20.8 | | |
|  | | | | | | Amibara | | | | | 0 | | | | 2 | 30 | | 27 | | 59 | | | 38.3 | | |
| Negative | | | | | | Awash Fentale | | | | | 13 | | | | 7 | 0 | | 0 | | 20 | | | 13.0 | | |
|  | | | | | | Amibara | | | | | 0 | | | | 0 | 27 | | 13 | | 40 | | | 26.0 | | |
| Neutral | | | | | | Awash Fentale | | | | | 0 | | | | 0 | 0 | | 0 | | 0 | | | 0.0 | | |
|  | | | | | | Amibara | | | | | 0 | | | | 0 | 2 | | 1 | | 3 | | | 1.9 | | |
| Do think *P.juliflora* will have impacts on biodiversity? | | | | | | | | | | | | | | |  |  | |  | |  | | |  | | |
| Agree | | | | Awash Fentale | | | | | | | 17 | | | | 19 | 0 | | 0 | | 36 | | | 23.4 | | |
|  | | | | Amibara | | | | | | | 0 | | | | 1 | 29 | | 26 | | 56 | | | 36.4 | | |
| Disagree | | | | Awash Fentale | | | | | | | 5 | | | | 11 | 0 | | 0 | | 16 | | | 10.4 | | |
|  | | | | Amibara | | | | | | | 0 | | | | 1 | 29 | | 15 | | 45 | | | 29.2 | | |
| Neutral | | | | Awash Fentale | | | | | | | 0 | | | | 0 | 0 | | 0 | | 0 | | | 0.0 | | |
|  | | | | Amibara | | | | | | | 0 | | | | 0 | 1 | | 0 | | 1 | | | 0.6 | | |
| What kinds of impacts biodiversity on biodiversity? | | | | | | | | | | | | | | |  |  | |  | |  | | |  | | |
| Positive | | | | | Awash Fentale | | | | | | 0 | | | | 10 | 0 | | 0 | | 10 | | | 6.5 | | |
|  | | | | | Amibara | | | | | | 0 | | | | 1 | 13 | | 3 | | 17 | | | 11.0 | | |
| Negative | | | | | Awash Fentale | | | | | | 22 | | | | 20 | 0 | | 0 | | 42 | | | 27.3 | | |
|  | | | | | Amibara | | | | | | 0 | | | | 1 | 46 | | 38 | | 85 | | | 55.2 | | |
| What type of biodiversity affected by *P.juliflora*? | | | | | | | | | | | | | | |  |  | |  | |  | | |  | | |
| Livestock | | | | | Awash Fentale | | | | | | 16 | | | | 26 | 0 | | 0 | | 42 | | | 27.3 | | |
|  | | | | | Amibara | | | | | | 0 | | | | 2 | 44 | | 23 | | 69 | | | 44.8 | | |
| Plants | | | | | Awash Fentale | | | | | | 6 | | | | 4 | 0 | | 0 | | 10 | | | 6.5 | | |
|  | | | | | Amibara | | | | | | 0 | | | | 0 | 14 | | 17 | | 31 | | | 20.1 | | |
| Human beings | | | | | Awash Fentale | | | | | | 0 | | | | 0 | 0 | | 0 | | 0 | | | 0.0 | | |
|  | | | | | Amibara | | | | | | 0 | | | | 0 | 1 | | 1 | | 2 | | | 1.3 | | |
| Which will be the most affected biodiversity by *P.juliflora*? | | | | | | | | | | | | | | |  |  | |  | |  | | |  | | |
| Livestock | | | | | Awash Fentale | | | | | | 20 | | | | 27 | 0 | | 0 | | 47 | | | 30.5 | | |
|  | | | | | Amibara | | | | | | 0 | | | | 2 | 51 | | 16 | | 69 | | | 44.8 | | |
| Plants | | | | | Awash Fentale | | | | | | 2 | | | | 3 | 0 | | 0 | | 5 | | | 3.2 | | |
|  | | | | | Amibara | | | | | | 0 | | | | 0 | 5 | | 24 | | 29 | | | 18.8 | | |
| Human beings | | | | | Awash Fentale | | | | | | 0 | | | | 0 | 0 | | 0 | | 0 | | | 0.0 | | |
|  | | | | | Amibara | | | | | | 0 | | | | 0 | 0 | | 1 | | 1 | | | 0.6 | | |
| Wild animals | | | | | Awash Fentale | | | | | | 0 | | | | 0 | 0 | | 0 | | 0 | | | 0.0 | | |
|  | | | | | Amibara | | | | | | 0 | | | | 0 | 3 | | 0 | | 3 | | | 1.9 | | |
| How is *P.juliflora* encroachment influencing livestock productivity in your site? | | | | | | | | | | | | | | | | | | | | | | | | | |
| Cause for feed shortage | | | | | Awash Fentale | | | | | | 13 | | | | 27 | 0 | | 0 | | 40 | | | 26.0 | | |
|  | | | | | Amibara | | | | | | 0 | | | | 0 | 40 | | 28 | | 68 | | | 44.2 | | |
| Cause of diseases | | | | | Awash Fentale | | | | | | 6 | | | | 2 | 0 | | 0 | | 8 | | | 5.2 | | |
|  | | | | | Amibara | | | | | | 0 | | | | 0 | 4 | | 6 | | 10 | | | 6.5 | | |
| Cause water deficit | | | | | Awash Fentale | | | | | | 0 | | | | 1 | 0 | | 0 | | 1 | | | 0.6 | | |
|  | | | | | Amibara | | | | | | 0 | | | | 2 | 2 | | 0 | | 4 | | | 2.6 | | |
| Blocking pass way | | | | | Awash Fentale | | | | | | 0 | | | | 0 | 0 | | 0 | | 0 | | | 0.0 | | |
|  | | | | | Amibara | | | | | | 0 | | | | 0 | 13 | | 7 | | 20 | | | 13.0 | | |
| Others | | | | | Awash Fentale | | | | | | 3 | | | | 0 | 0 | | 0 | | 3 | | | 1.9 | | |
|  | | | | | Amibara | | | | | | 0 | | | | 0 | 0 | | 0 | | 0 | | | 0.0 | | |
| Do *P.juliflora* impacts on plants? | | | | | | | | | | | | | | |  |  | |  | |  | | |  | | |
| Yes | | | | | | Awash Fentale | | | | | 9 | | | | 23 | 0 | | 0 | | 32 | | | 20.8 | | |
|  | | | | | | Amibara | | | | | 0 | | | | 2 | 30 | | 27 | | 59 | | | 38.3 | | |
| No | | | | | | Awash Fentale | | | | | 13 | | | | 7 | 0 | | 0 | | 20 | | | 13.0 | | |
|  | | | | | | Amibara | | | | | 0 | | | | 0 | 27 | | 13 | | 40 | | | 26.0 | | |
| I do not know | | | | | | Awash Fentale | | | | | 0 | | | | 0 | 0 | | 0 | | 0 | | | 0.0 | | |
|  | | | | | | Amibara | | | | | 0 | | | | 0 | 2 | | 1 | | 3 | | | 1.9 | | |
| Do think *P.juliflora* will have impacts on biodiversity? | | | | | | | | | | | | | | |  |  | |  | |  | | |  | | |
| It takes water away | | | Awash Fentale | | | | | | | | 17 | | | | 19 | 0 | | 0 | | 36 | | | 23.4 | | |
|  | | | Amibara | | | | | | | | 0 | | | | 1 | 29 | | 26 | | 56 | | | 36.4 | | |
| It takes nutrient | | | Awash Fentale | | | | | | | | 5 | | | | 11 | 0 | | 0 | | 16 | | | 10.4 | | |
|  | | | Amibara | | | | | | | | 0 | | | | 1 | 29 | | 15 | | 45 | | | 29.2 | | |
| It takes sunlight | | | Awash Fentale | | | | | | | | 0 | | | | 0 | 0 | | 0 | | 0 | | | 0.0 | | |
|  | | | Amibara | | | | | | | | 0 | | | | 0 | 1 | | 0 | | 1 | | | 0.6 | | |
| What kinds of impacts *P.juliflora* on biodiversity? | | | | | | | | | | | | | | |  |  | |  | |  | | |  | | |
| Positive | | | Awash Fentale | | | | | | | | 0 | | | | 10 | 0 | | 0 | | 10 | | | 6.5 | | |
|  | | | Amibara | | | | | | | | 0 | | | | 1 | 13 | | 3 | | 17 | | | 11.0 | | |
| Negative | | | Awash Fentale | | | | | | | | 22 | | | | 20 | 0 | | 0 | | 42 | | | 27.3 | | |
|  | | | Amibara | | | | | | | | 0 | | | | 1 | 46 | | 38 | | 85 | | | 55.2 | | |
| Which will be the most affected biodiversity? | | | | | | | | | | | | | | |  |  | |  | |  | | |  | | |
| Livestock | | | | | Awash Fentale | | | | | | 20 | | | | 27 | 0 | | 0 | | 47 | | | 30.5 | | |
|  | | | | | Amibara | | | | | | 0 | | | | 2 | 51 | | 16 | | 69 | | | 44.8 | | |
| Plants | | | | | Awash Fentale | | | | | | 2 | | | | 3 | 0 | | 0 | | 5 | | | 3.2 | | |
|  | | | | | Amibara | | | | | | 0 | | | | 0 | 5 | | 24 | | 29 | | | 18.8 | | |
| Human beings | | | | | Awash Fentale | | | | | | 0 | | | | 0 | 0 | | 0 | | 0 | | | 0.0 | | |
|  | | | | | Amibara | | | | | | 0 | | | | 0 | 0 | | 1 | | 1 | | | 0.6 | | |
| Wild animals | | | | | Awash Fentale | | | | | | 0 | | | | 0 | 0 | | 0 | | 0 | | | 0.0 | | |
|  | | | | | Amibara | | | | | | 0 | | | | 0 | 3 | | 0 | | 3 | | | 1.9 | | |
| Does *P.juliflora* influence crop harvest negatively? | | | | | | | | | | | | | | | |  | |  | |  | | |  | | |
| Yes | | | | | Awash Fentale | | | | | | 22 | | | | 26 | 0 | | 1 | | 48 | | | 31.2 | | |
|  | | | | | Amibara | | | | | | 0 | | | | 2 | 46 | | 26 | | 74 | | | 48.1 | | |
| No | | | | | Awash Fentale | | | | | | 0 | | | | 2 | 0 | | 0 | | 2 | | | 1.3 | | |
|  | | | | | Amibara | | | | | | 0 | | | | 0 | 12 | | 3 | | 15 | | | 9.7 | | |
| I do not know | | | | | Awash Fentale | | | | | | 0 | | | | 2 | 0 | | 0 | | 2 | | | 1.3 | | |
|  | | | | | Amibara | | | | | | 0 | | | | 0 | 1 | | 11 | | 12 | | | 7.8 | | |
| Negative effects of *P.juliflora* on crops? | | | | | | | | | | | | | | |  |  | |  | |  | | |  | | |
| It takes water away | | | | | | Awash Fentale | | | | | 11 | | | | 21 | 0 | | 0 | | 32 | | | 20.8 | | |
|  | | | | | | Amibara | | | | | 0 | | | | 2 | 18 | | 8 | | 28 | | | 18.2 | | |
| It takes nutrients | | | | | | Awash Fentale | | | | | 6 | | | | 2 | 0 | | 0 | | 8 | | | 5.2 | | |
|  | | | | | | Amibara | | | | | 0 | | | | 0 | 13 | | 6 | | 19 | | | 12.3 | | |
| Share sunlight | | | | | | Awash Fentale | | | | | 4 | | | | 2 | 0 | | 0 | | 6 | | | 3.9 | | |
|  | | | | | | Amibara | | | | | 0 | | | | 0 | 10 | | 17 | | 27 | | | 17.5 | | |
| Block roads | | | | | | Awash Fentale | | | | | 0 | | | | 4 | 0 | | 0 | | 4 | | | 2.6 | | |
|  | | | | | | Amibara | | | | | 0 | | | | 0 | 18 | | 10 | | 28 | | | 18.2 | | |
| Others | | | | | | Awash Fentale | | | | | 1 | | | | 1 | 0 | | 0 | | 2 | | | 1.3 | | |
|  | | | | | | Amibara | | | | | 0 | | | | 0 | 0 | | 0 | | 0 | | | 0.0 | | |
| Negative effects of *P.juliflora* on rangeland? | | | | | | | | | | | | | | | | | | | | | | | | | |
| It takes water away | | | | | | Awash Fentale | | | | | 13 | | | | 19 | 0 | | 0 | | 32 | | | 20.8 | | |
|  | | | | | | Amibara | | | | | 0 | | | | 1 | 21 | | 13 | | 35 | | | 22.7 | | |
| It takes nutrients | | | | | | Awash Fentale | | | | | 7 | | | | 5 | 0 | | 0 | | 12 | | | 7.8 | | |
|  | | | | | | Amibara | | | | | 0 | | | | 1 | 10 | | 4 | | 15 | | | 9.7 | | |
| Share sunlight | | | | | | Awash Fentale | | | | | 1 | | | | 4 | 0 | | 0 | | 5 | | | 3.2 | | |
|  | | | | | | Amibara | | | | | 0 | | | | 0 | 15 | | 2 | | 17 | | | 11.0 | | |
| Block roads | | | | | | Awash Fentale | | | | | 0 | | | | 2 | 0 | | 0 | | 2 | | | 1.3 | | |
|  | | | | | | Amibara | | | | | 0 | | | | 0 | 13 | | 22 | | 35 | | | 22.7 | | |
| Others | | | | | | Awash Fentale | | | | | 1 | | | | 0 | 0 | | 0 | | 1 | | | 0.6 | | |
|  | | | | | | Amibara | | | | | 0 | | | | 0 | 0 | | 0 | | 0 | | | 0.0 | | |
| Did you see/heard animals dying eating *P.juliflora*? | | | | | | | | | | |  | | | |  |  | |  | |  | | |  | | |
| Yes | | | | | Awash Fentale | | | | | | 22 | | | | 30 | 0 | | 0 | | 52 | | | 33.8 | | |
|  | | | | | Amibara | | | | | | 0 | | | | 2 | 59 | | 41 | | 102 | | | 66.2 | | |
| What impacts *P.juliflora* on human beings? | | | | | | | | | | | | | | | | | | | | | | | | | |
| Allergic pollen on skin | | | | | Awash Fentale | | | | | | 1 | | | | 11 | 0 | | 0 | | 12 | | | 7.8 | | |
|  | | | | | Amibara | | | | | | 0 | | | | 1 | 3 | | 2 | | 6 | | | 3.9 | | |
| Long thorn are piercing and become toxic | | | | | Awash Fentale | | | | | | 15 | | | | 15 | 0 | | 0 | | 30 | | | 19.5 | | |
|  | | | | | Amibara | | | | | | 0 | | | | 0 | 35 | | 11 | | 46 | | | 29.9 | | |
| Block roads | | | | | Awash Fentale | | | | | | 6 | | | | 4 | 0 | | 0 | | 10 | | | 6.5 | | |
|  | | | | | Amibara | | | | | | 0 | | | | 1 | 21 | | 28 | | 50 | | | 32.5 | | |
| Which part of *P.juliflora* killing animals? | | | | | | | | | | | | | | | | | | | | | | | | | |
| Leaf | Awash Fentale | | | | | | | | | | 10 | | | | 28 | 0 | | 0 | | 38 | | | 24.7 | | |
|  | Amibara | | | | | | | | | | 0 | | | | 2 | 25 | | 22 | | 49 | | | 31.8 | | |
| Thorns | Awash Fentale | | | | | | | | | | 0 | | | | 0 | 0 | | 0 | | 0 | | | 0.0 | | |
|  | Amibara | | | | | | | | | | 0 | | | | 0 | 3 | | 1 | | 4 | | | 2.6 | | |
| Fruits | Awash Fentale | | | | | | | | | | 0 | | | | 0 | 0 | | 0 | | 0 | | | 0.0 | | |
|  | Amibara | | | | | | | | | | 0 | | | | 0 | 1 | | 1 | | 2 | | | 1.3 | | |
| Seeds | Awash Fentale | | | | | | | | | | 10 | | | | 1 | 0 | | 0 | | 11 | | | 7.1 | | |
|  | Amibara | | | | | | | | | | 0 | | | | 0 | 28 | | 17 | | 45 | | | 29.2 | | |
| Plant juice | Awash Fentale | | | | | | | | | | 2 | | | | 1 | 0 | | 0 | | 3 | | | 1.9 | | |
|  | Amibara | | | | | | | | | | 0 | | | | 0 | 2 | | 0 | | 2 | | | 1.3 | | |
| Appendix 13. Perceptions of communities towards recurrent status, effects on LULCC, palatable parts and its effects of *P.juliflora* in South Afar Region | | | | | | | | | | | | | | | | | | | | | | | | | |
| What do you think is the Afar people attitude for *P.juliflora*? | | | | | | | | | | | | | | | | | | | | | | | | | |
| t is as beneficial species | | | Awash Fentale | | | | | | | | 0 | | | | 3 | 0 | | 0 | | 3 | | | 1.9 | | |
|  | | | Amibara | | | | | | | | 0 | | | | 0 | 6 | | 0 | | 6 | | | 3.9 | | |
| It is as deleterious species | | | Awash Fentale | | | | | | | | 22 | | | | 27 | 0 | | 0 | | 49 | | | 31.8 | | |
|  | | | Amibara | | | | | | | | 0 | | | | 2 | 52 | | 39 | | 93 | | | 60.4 | | |
| Others | | | Awash Fentale | | | | | | | | 0 | | | | 0 | 0 | | 0 | | 0 | | | 0.0 | | |
|  | | | Amibara | | | | | | | | 0 | | | | 0 | 1 | | 1 | | 2 | | | 1.3 | | |
| Is spread of *P.juliflora* increasing or declining? | | | | | | | | | | | | | | | | | | | | | | | | | |
| Yes | | | | Awash Fentale | | | | | | | 21 | | | | 30 | 0 | | 0 | | 51 | | | 33.1 | | |
|  | | | | Amibara | | | | | | | 0 | | | | 2 | 58 | | 38 | | 98 | | | 63.6 | | |
| No | | | | Awash Fentale | | | | | | | 1 | | | | 0 | 0 | | 0 | | 1 | | | 0.6 | | |
|  | | | | Amibara | | | | | | | 0 | | | | 0 | 1 | | 2 | | 3 | | | 1.9 | | |
| What changes are observing in livestock holdings during the last decade? | | | | | | | | | | | | | | | | | | | | | | | | | |
| Yes | | | | | | Awash Fentale | | | | | 18 | | | | 30 | 0 | | 0 | | 48 | | | 31.2 | | |
|  | | | | | | Amibara | | | | | 0 | | | | 2 | 59 | | 40 | | 101 | | | 65.6 | | |
| No | | | | | | Awash Fentale | | | | | 4 | | | | 0 | 0 | | 0 | | 4 | | | 2.6 | | |
|  | | | | | | Amibara | | | | | 0 | | | | 0 | 0 | | 0 | | 0 | | | 0.0 | | |
| What are the causes for the changes of the physical conditions of livestock? | | | | | | | | | | | | | | | | | | | | | | | | | |
| Shortage of rainfall | | | | | | Awash Fentale | | | | | 13 | | | | 16 | 0 | | 0 | | 29 | | | 18.8 | | |
|  | | | | | | Amibara | | | | | 0 | | | | 0 | 27 | | 26 | | 53 | | | 34.4 | | |
| Agricultural expansion | | | | | | Awash Fentale | | | | | 1 | | | | 0 | 0 | | 0 | | 1 | | | 0.6 | | |
|  | | | | | | Amibara | | | | | 0 | | | | 0 | 0 | | 2 | | 2 | | | 1.3 | | |
| Invasion of *P.juliflora* | | | | | | Awash Fentale | | | | | 4 | | | | 14 | 0 | | 0 | | 18 | | | 11.7 | | |
|  | | | | | | Amibara | | | | | 0 | | | | 2 | 30 | | 10 | | 42 | | | 27.3 | | |
| Diseases | | | | | | Awash Fentale | | | | | 1 | | | | 0 | 0 | | 0 | | 1 | | | 0.6 | | |
|  | | | | | | Amibara | | | | | 0 | | | | 0 | 2 | | 2 | | 4 | | | 2.6 | | |
| Others | | | | | | Awash Fentale | | | | | 3 | | | | 0 | 0 | | 0 | | 3 | | | 1.9 | | |
|  | | | | | | Amibara | | | | | 0 | | | | 0 | 0 | | 0 | | 0 | | | 0.0 | | |
| How the changes above are responsible? | | | | | | | | | | | | | | | | | | | | | | | | | |
| In terms of grazing land encroachment | | | | | | | Awash Fentale | | | | 18 | | | | 11 | 0 | | 0 | | 29 | | | 18.8 | | |
|  | | | | | | | Amibara | | | | 0 | | | | 0 | 22 | | 26 | | 48 | | | 31.2 | | |
| Loss quality and amount of feeds for livestock | | | | | | | Awash Fentale | | | | 0 | | | | 2 | 0 | | 0 | | 2 | | | 1.3 | | |
|  | | | | | | | Amibara | | | | 0 | | | | 0 | 6 | | 6 | | 12 | | | 7.8 | | |
| In terms declining of livestock production and productivity | | | | | | | Awash Fentale | | | | 4 | | | | 17 | 0 | | 0 | | 21 | | | 13.6 | | |
|  | | | | | | | Amibara | | | | 0 | | | | 2 | 28 | | 3 | | 33 | | | 21.4 | | |
| Loss of drinking water for livestock | | | | | | | Awash Fentale | | | | 0 | | | | 0 | 0 | | 0 | | 0 | | | 0.0 | | |
|  | | | | | | | Amibara | | | | 0 | | | | 0 | 2 | | 5 | | 7 | | | 4.5 | | |
| Others | | | | | | | Awash Fentale | | | | 0 | | | | 0 | 0 | | 0 | | 0 | | | 0.0 | | |
|  | | | | | | | Amibara | | | | 0 | | | | 0 | 1 | | 0 | | 1 | | | 0.6 | | |
| How grazing land availability after introduction of *P.juliflora*? | | | | | | | | | | | | | | | | | | | | | | | | | |
| Declining | | | | | | | Awash Fentale | | | | 22 | | | | 28 | 0 | | 0 | | 50 | | | 32.5 | | |
|  | | | | | | | Amibara | | | | 0 | | | | 2 | 52 | | 38 | | 92 | | | 59.7 | | |
| Increasing | | | | | | | Awash Fentale | | | | 0 | | | | 2 | 0 | | 0 | | 2 | | | 1.3 | | |
|  | | | | | | | Amibara | | | | 0 | | | | 0 | 7 | | 1 | | 8 | | | 5.2 | | |
| Which LULC converted last 10 years? | | | | | | | | | | | | | | | | | | | | | | | | | |
| Acacia woodland to *P.juliflora* thicket | | | | | | | Awash Fentale | | | | 8 | | | | 12 | 0 | | 0 | | 20 | | | 13.0 | | |
|  | | | | | | | Amibara | | | | 0 | | | | 0 | 23 | | 31 | | 54 | | | 35.1 | | |
| Acacia woodland to settlement | | | | | | | Awash Fentale | | | | 0 | | | | 0 | 0 | | 0 | | 0 | | | 0.0 | | |
|  | | | | | | | Amibara | | | | 0 | | | | 1 | 1 | | 0 | | 2 | | | 1.3 | | |
| Acacia woodland to grass land | | | | | | | Awash Fentale | | | | 1 | | | | 0 | 0 | | 0 | | 1 | | | 0.6 | | |
|  | | | | | | | Amibara | | | | 0 | | | | 0 | 0 | | 0 | | 0 | | | 0.0 | | |
| Grass land to *P.juliflora* | | | | | | | Awash Fentale | | | | 7 | | | | 17 | 0 | | 0 | | 24 | | | 15.6 | | |
|  | | | | | | | Amibara | | | | 0 | | | | 1 | 34 | | 7 | | 42 | | | 27.3 | | |
| Bare land with *P.juliflora* thicket | | | | | | | Awash Fentale | | | | 0 | | | | 0 | 0 | | 0 | | 0 | | | 0.0 | | |
|  | | | | | | | Amibara | | | | 0 | | | | 0 | 0 | | 2 | | 2 | | | 1.3 | | |
| Farm land to water body | | | | | | | Awash Fentale | | | | 0 | | | | 1 | 0 | | 0 | | 1 | | | 0.6 | | |
|  | | | | | | | Amibara | | | | 0 | | | | 0 | 0 | | 0 | | 0 | | | 0.0 | | |
| Water body to *P.juliflora* thicket | | | | | | | Awash Fentale | | | | 4 | | | | 0 | 0 | | 0 | | 4 | | | 2.6 | | |
|  | | | | | | | Amibara | | | | 0 | | | | 0 | 1 | | 0 | | 1 | | | 0.6 | | |
| Others | | | | | | | Awash Fentale | | | | 1 | | | | 0 | 0 | | 0 | | 1 | | | 0.6 | | |
|  | | | | | | | Amibara | | | | 0 | | | | 0 | 0 | | 0 | | 0 | | | 0.0 | | |
| What are the causes for LULC conversions? | | | | | | | | | | | | | | | | | | | | | | | | | |
| Livestock production beyond the carrying capacity/overgrazing | | | | | | | | Awash Fentale | | | | | | 7 | 11 | 0 | | 0 | | 17 | | | 11.0 | | |
|  | | | | | | | | Amibara | | | | | | 0 | 2 | 19 | | 4 | | 25 | | | 16.2 | | |
| Farm land expansion | | | | | | | | Awash Fentale | | | | | | 3 | 1 | 0 | | 0 | | 4 | | | 2.6 | | |
|  | | | | | | | | Amibara | | | | | | 0 | 0 | 0 | | 0 | | 0 | | | 0.0 | | |
| Anthropogenic | | | | | | | | Awash Fentale | | | | | | 0 | 0 | 0 | | 0 | | 0 | | | 0.0 | | |
|  | | | | | | | | Amibara | | | | | | 0 | 0 | 1 | | 0 | | 1 | | | 0.6 | | |
| Invasion of *P.juliflora* | | | | | | | | Awash Fentale | | | | | | 8 | 16 | 0 | | 0 | | 24 | | | 15.6 | | |
|  | | | | | | | | Amibara | | | | | | 0 | 0 | 26 | | 16 | | 42 | | | 27.3 | | |
| Moisture stress | | | | | | | | Awash Fentale | | | | | | 2 | 0 | 0 | | 0 | | 2 | | | 1.3 | | |
|  | | | | | | | | Amibara | | | | | | 0 | 0 | 0 | | 0 | | 0 | | | 0.0 | | |
| Drought/shortage of rainfall | | | | | | | | Awash Fentale | | | | | | 1 | 0 | 0 | | 0 | | 1 | | | 0.6 | | |
|  | | | | | | | | Amibara | | | | | | 0 | 0 | 7 | | 2 | | 9 | | | 5.8 | | |
| Toxic effects of *P.juliflora* | | | | | | | | Awash Fentale | | | | | | 0 | 2 | 0 | | 0 | | 2 | | | 1.3 | | |
|  | | | | | | | | Amibara | | | | | | 0 | 0 | 6 | | 18 | | 24 | | | 15.6 | | |
| Are wild lives under *P.juliflora*? | | | | | | | | | | | | | | | | | | | | | | | | | |
| Yes | | | | | | | Awash Fentale | | | | 13 | | | | 23 | 0 | | 0 | | 36 | | | 23.4 | | |
|  | | | | | | | Amibara | | | | 0 | | | | 2 | 46 | | 33 | | 81 | | | 52.6 | | |
| No | | | | | | | Awash Fentale | | | | 8 | | | | 7 | 0 | | 0 | | 15 | | | 9.7 | | |
|  | | | | | | | Amibara | | | | 0 | | | | 0 | 13 | | 7 | | 20 | | | 13.0 | | |
| In which season do animals feed on *P.juliflora*? | | | | | | | | | | | | | | | | | | | | | | | | | |
| Dry | | | | | | | Awash Fentale | | | | 21 | | | | 27 | 0 | | 0 | | 48 | | | 31.2 | | |
|  | | | | | | | Amibara | | | | 0 | | | | 2 | 54 | | 39 | | 95 | | | 61.7 | | |
| Wet | | | | | | | Awash Fentale | | | | 1 | | | | 3 | 0 | | 0 | | 4 | | | 2.6 | | |
|  | | | | | | | Amibara | | | | 0 | | | | 0 | 5 | | 1 | | 6 | | | 3.9 | | |
| Which part of *P.juliflora* palatable by animals? | | | | | | | | | | | | | | | | | | | | | | | | | |
| Leaf | | | | | | Awash Fentale | | | | | 0 | | | | 4 | 0 | | 0 | | 4 | | | 2.6 | | |
|  | | | | | | Amibara | | | | | 0 | | | | 0 | 6 | | 18 | | 24 | | | 15.6 | | |
| Pod/seed | | | | | | Awash Fentale | | | | | 22 | | | | 26 | 0 | | 0 | | 48 | | | 31.2 | | |
|  | | | | | | Amibara | | | | | 0 | | | | 2 | 47 | | 22 | | 71 | | | 46.1 | | |
| Bark | | | | | | Awash Fentale | | | | | 0 | | | | 0 | 0 | | 0 | | 0 | | | 0.0 | | |
|  | | | | | | Amibara | | | | | 0 | | | | 0 | 2 | | 0 | | 2 | | | 1.3 | | |
| Flower | | | | | | Awash Fentale | | | | | 0 | | | | 0 | 0 | | 0 | | 0 | | | 0.0 | | |
|  | | | | | | Amibara | | | | | 0 | | | | 0 | 4 | | 0 | | 4 | | | 2.6 | | |
| Which part of *P.juliflora* is eaten by cattle? | | | | | | | | | | | | | | | | | | | | | | | | | |
| Leaf | | | | | | | Awash Fentale | | | | 0 | | | | 3 | 0 | | 0 | | 3 | | | 1.9 | | |
|  | | | | | | | Amibara | | | | 0 | | | | 0 | 8 | | 20 | | 28 | | | 18.2 | | |
| Roots | | | | | | | Awash Fentale | | | | 1 | | | | 0 | 0 | | 0 | | 1 | | | 0.6 | | |
|  | | | | | | | Amibara | | | | 0 | | | | 0 | 4 | | 1 | | 5 | | | 3.2 | | |
| Pod/seed | | | | | | | Awash Fentale | | | | 21 | | | | 27 | 0 | | 0 | | 48 | | | 31.2 | | |
|  | | | | | | | Amibara | | | | 0 | | | | 2 | 44 | | 18 | | 64 | | | 41.6 | | |
| Flower | | | | | | | Awash Fentale | | | | 0 | | | | 0 | 0 | | 0 | | 0 | | | 0.0 | | |
|  | | | | | | | Amibara | | | | 0 | | | | 0 | 3 | | 1 | | 4 | | | 2.6 | | |
| Which part of *P.juliflora* is eaten by sheep? | | | | | | | | | | | | | | | | | | | | | | | | | |
| Pod/seed | | | | | | Awash Fentale | | | | | 22 | | | | 28 | 0 | | 0 | | 50 | | | 32.5 | | |
|  | | | | | | Amibara | | | | | 0 | | | | 2 | 51 | | 31 | | 84 | | | 54.5 | | |
| Leaf | | | | | | Awash Fentale | | | | | 0 | | | | 0 | 0 | | 0 | | 1 | | | 0.6 | | |
|  | | | | | | Amibara | | | | | 0 | | | | 0 | 2 | | 8 | | 10 | | | 6.5 | | |
| Bark | | | | | | Awash Fentale | | | | | 0 | | | | 1 | 0 | | 0 | | 1 | | | 0.6 | | |
|  | | | | | | Amibara | | | | | 0 | | | | 0 | 2 | | 1 | | 3 | | | 1.9 | | |
| Flower | | | | | | Awash Fentale | | | | | 0 | | | | 1 | 2 | | 1 | | 4 | | | 2.6 | | |
|  | | | | | | Amibara | | | | | 0 | | | | 0 | 4 | | 0 | | 4 | | | 2.6 | | |
| Which part of *P.juliflora* is eaten by goats? | | | | | | | | | | | | | | | | | | | | | | | | | |
| Pod/seed | | | | | | Awash Fentale | | | | | 22 | | | | 30 | 0 | | 0 | | 52 | | | 33.8 | | |
|  | | | | | | Amibara | | | | | 0 | | | | 2 | 49 | | 32 | | 83 | | | 53.9 | | |
| Leaf | | | | | | Awash Fentale | | | | | 0 | | | | 0 | 0 | | 0 | | 0 | | | 0.0 | | |
|  | | | | | | Amibara | | | | | 0 | | | | 0 | 3 | | 6 | | 9 | | | 5.8 | | |
| Flower | | | | | | Awash Fentale | | | | | 0 | | | | 0 | 0 | | 0 | | 0 | | | 0.0 | | |
|  | | | | | | Amibara | | | | | 0 | | | | 0 | 7 | | 2 | | 9 | | | 5.8 | | |
| Which part of *P.juliflora* is eaten by camels? | | | | | | | | | | | | | | | | | | | | | | | | | |
| Pod/seed | | | | | | | Awash Fentale | | | | 22 | | | | 30 | 0 | | 0 | | 52 | | | 33.8 | | |
|  | | | | | | | Amibara | | | | 0 | | | | 2 | 51 | | 20 | | 77 | | | 50.0 | | |
| Leaf | | | | | | | Awash Fentale | | | | 0 | | | | 0 | 0 | | 0 | | 0 | | | 0.0 | | |
|  | | | | | | | Amibara | | | | 0 | | | | 0 | 3 | | 12 | | 15 | | | 9.7 | | |
| Roots | | | | | | | Awash Fentale | | | | 0 | | | | 0 | 0 | | 0 | | 0 | | | 0.0 | | |
|  | | | | | | | Amibara | | | | 0 | | | | 0 | 1 | | 0 | | 1 | | | 0.6 | | |
| Flower | | | | | | | Awash Fentale | | | | 0 | | | | 0 | 0 | | 0 | | 0 | | | 0.0 | | |
|  | | | | | | | Amibara | | | | 0 | | | | 0 | 4 | | 4 | | 8 | | | 5.2 | | |
| Which part of *P.juliflora* is eaten by donkeys? | | | | | | | | | | | | | | | | | | | | | | | | | |
| Pod/seed | | | | | | | Awash Fentale | | | | 22 | | | | 29 | 0 | | 0 | | 51 | | | 33.1 | | |
|  | | | | | | | Amibara | | | | 0 | | | | 2 | 56 | | 39 | | 97 | | | 63.0 | | |
| Bark | | | | | | | Awash Fentale | | | | 0 | | | | 0 | 0 | | 0 | | 0 | | | 0.0 | | |
|  | | | | | | | Amibara | | | | 0 | | | | 0 | 1 | | 0 | | 1 | | | 0.6 | | |
| Leaf | | | | | | | Awash Fentale | | | | 0 | | | | 0 | 0 | | 0 | | 0 | | | 0.0 | | |
|  | | | | | | | Amibara | | | | 0 | | | | 0 | 1 | | 1 | | 2 | | | 1.3 | | |
| Roots | | | | | | | Awash Fentale | | | | 0 | | | | 1 | 0 | | 0 | | 1 | | | 0.6 | | |
|  | | | | | | | Amibara | | | | 0 | | | | 0 | 1 | | 0 | | 1 | | | 0.6 | | |
| In which season do you observe negative effects on your livestock? | | | | | | | | | | | | | | | | | | | | | | | | | |
| Dry | | | | | Awash Fentale | | | | | | 21 | | | | 26 | 0 | | 0 | | 47 | | | 30.5 | | |
|  | | | | | Amibara | | | | | | 0 | | | | 2 | 52 | | 37 | | 91 | | | 59.1 | | |
| Wet | | | | | Awash Fentale | | | | | | 1 | | | | 4 | 0 | | 0 | | 5 | | | 3.2 | | |
|  | | | | | Amibara | | | | | | 0 | | | | 0 | 7 | | 3 | | 10 | | | 6.5 | | |
| Did you observed the concentration of *P.juliflora* in taking of animals? | | | | | | | | | | | | | | | | | | | | | | | | | |
| Yes | | | | Awash Fentale | | | | | | | 21 | | | | 30 | 0 | | 0 | | 51 | | | 33.1 | | |
|  | | | | Amibara | | | | | | | 1 | | | | 3 | 60 | | 41 | | 105 | | | 68.2 | | |
| No | | | | Awash Fentale | | | | | | | 1 | | | | 0 | 0 | | 0 | | 1 | | | 0.6 | | |
|  | | | | Amibara | | | | | | | 0 | | | | 0 | 0 | | 0 | | 0 | | | 0.0 | | |
| If yes, which animal domestic animal most affected by *P.juliflora?* | | | | | | | | | | | | | | | | | | | | | | | | | |
| Cattle | | | | Awash Fentale | | | | | | | 21 | | | | 20 | 0 | | 0 | | 41 | | 26.6 | | | |
|  | | | | Amibara | | | | | | | 0 | | | | 0 | 33 | | 3 | | 36 | | 23.4 | | | |
| Camels | | | | Awash Fentale | | | | | | | 0 | | | | 0 | 0 | | 0 | | 0 | | 0.0 | | | |
|  | | | | Amibara | | | | | | | 0 | | | | 0 | 1 | | 3 | | 4 | | 2.6 | | | |
| Goats | | | | Awash Fentale | | | | | | | 0 | | | | 6 | 0 | | 0 | | 6 | | 3.9 | | | |
|  | | | | Amibara | | | | | | | 0 | | | | 1 | 18 | | 28 | | 47 | | 30.5 | | | |
| Sheep | | | | Awash Fentale | | | | | | | 1 | | | | 4 | 0 | | 0 | | 5 | | 3.2 | | | |
|  | | | | Amibara | | | | | | | 0 | | | | 1 | 7 | | 6 | | 14 | | 9.1 | | | |
| How does the intake of *P.juliflora* compare with other animal feed? | | | | | | | | | | | | | | | | | | | | | | | | | |
| Mostly other natural pasture | | | | | Awash Fentale | | | | | | 8 | | | | 11 | 0 | | 0 | | 19 | | | 12.3 | | |
|  | | | | | Amibara | | | | | | 0 | | | | 0 | 29 | | 7 | | 36 | | | 23.4 | | |
| Mostly other tree/shrub browsing | | | | | Awash Fentale | | | | | | 0 | | | | 0 | 0 | | 0 | | 0 | | | 0.0 | | |
|  | | | | | Amibara | | | | | | 0 | | | | 0 | 1 | | 6 | | 7 | | | 4.5 | | |
| Mostly *P.juliflora* browsing/feeds | | | | | Awash Fentale | | | | | | 7 | | | | 14 | 0 | | 0 | | 21 | | | 13.6 | | |
|  | | | | | Amibara | | | | | | 0 | | | | 2 | 12 | | 8 | | 22 | | | 14.3 | | |
| Other supplementary feeds | | | | | Awash Fentale | | | | | | 7 | | | | 4 | 0 | | 0 | | 11 | | | 7.1 | | |
|  | | | | | Amibara | | | | | | 0 | | | | 0 | 16 | | 19 | | 35 | | | 22.7 | | |
| Which feed do think and change the health of your animal? | | | | | | | | | | | | | | | | | | | | | | | | | |
| High concentration of feeds from natural pasture | | | | | | | | | Awash Fentale | | | | 11 | | 8 | 0 | | 0 | | 19 | | | 12.3 | | |
|  | | | | | | | | | Amibara | | | | 0 | | 0 | 15 | | 2 | | 17 | | | 11.0 | | |
| High concentration of feeds from other tree browsing | | | | | | | | | Awash Fentale | | | | 0 | | 2 | 0 | | 0 | | 2 | | | 1.3 | | |
|  | | | | | | | | | Amibara | | | | 0 | | 0 | 1 | | 3 | | 4 | | | 2.6 | | |
| High concentration of feeds from *P.juliflora* | | | | | | | | | Awash Fentale | | | | 10 | | 18 | 0 | | 0 | | 28 | | | 18.2 | | |
|  | | | | | | | | | Amibara | | | | 0 | | 2 | 33 | | 24 | | 59 | | | 38.3 | | |
| High concentration of feeds from other supplementary feeds | | | | | | | | | Awash Fentale | | | | 1 | | 1 | 0 | | 0 | | 2 | | | 1.3 | | |
|  | | | | | | | | | Amibara | | | | 0 | | 0 | 9 | | 11 | | 20 | | | 13.0 | | |
| Have you ever tried to control invasion of *P.juliflora*? | | | | | | | | | | | | | | | | | | | | | | | | | |
| Yes | | | | | | Awash Fentale | | | | | 22 | | | | 28 | 0 | | 0 | | 50 | | | 32.5 | | |
|  | | | | | | Amibara | | | | | 0 | | | | 2 | 44 | | 35 | | 81 | | | 52.6 | | |
| No | | | | | | Awash Fentale | | | | | 0 | | | | 1 | 0 | | 0 | | 1 | | | 0.6 | | |
|  | | | | | | Amibara | | | | | 0 | | | | 0 | 14 | | 5 | | 19 | | | 12.3 | | |
| Which measures are taken to control *P.juliflora*? | | | | | | | | | | | | | | | | | | | | | | | | | |
| Manage by utilization | | | | | | | Awash Fentale | | | | 7 | | | | 8 | 0 | | 0 | | 15 | | | 9.7 | | |
|  | | | | | | | Amibara | | | | 0 | | | | 0 | 13 | | 1 | | 14 | | | 9.1 | | |
| Mechanical control by cutting mature trees | | | | | | | Awash Fentale | | | | 11 | | | | 1 | 0 | | 0 | | 12 | | | 7.8 | | |
|  | | | | | | | Amibara | | | | 0 | | | | 0 | 5 | | 0 | | 5 | | | 3.2 | | |
| Mechanical control by uprooting mature trees | | | | | | | Awash Fentale | | | | 4 | | | | 0 | 0 | | 0 | | 4 | | | 2.6 | | |
|  | | | | | | | Amibara | | | | 0 | | | | 0 | 1 | | 0 | | 1 | | | 0.6 | | |
| Mechanical control by cutting juvenile stems | | | | | | | Awash Fentale | | | | 0 | | | | 1 | 0 | | 0 | | 1 | | | 0.6 | | |
|  | | | | | | | Amibara | | | | 0 | | | | 0 | 2 | | 4 | | 6 | | | 3.9 | | |
| Mechanical control by uprooting juvenile stems | | | | | | | Awash Fentale | | | | 0 | | | | 0 | 0 | | 0 | | 0 | | | 0.0 | | |
|  | | | | | | | Amibara | | | | 0 | | | | 0 | 1 | | 0 | | 1 | | | 0.6 | | |
| Fire | | | | | | | Awash Fentale | | | | 0 | | | | 19 | 0 | | 0 | | 19 | | | 12.3 | | |
|  | | | | | | | Amibara | | | | 0 | | | | 2 | 24 | | 25 | | 51 | | | 33.1 | | |
| Chemicals | | | | | | | Awash Fentale | | | | 0 | | | | 0 | 0 | | 0 | | 0 | | | 0.0 | | |
|  | | | | | | | Amibara | | | | 0 | | | | 0 | 12 | | 8 | | 20 | | | 13.0 | | |
| Control seed dispersal | | | | | | | Awash Fentale | | | | 0 | | | | 0 | 0 | | 0 | | 0 | | | 0.0 | | |
|  | | | | | | | Amibara | | | | 0 | | | | 0 | 0 | | 1 | | 1 | | | 0.6 | | |
| Using burned/used oil | | | | | | | Awash Fentale | | | | 0 | | | | 0 | 0 | | 0 | | 0 | | | 0.0 | | |
|  | | | | | | | Amibara | | | | 0 | | | | 0 | 0 | | 1 | | 1 | | | 0.6 | | |
| Is *P.juliflora* eradicated from Afar region? | | | | | | | | | | |  | | | |  |  | |  | |  | | |  | | |
| Yes | | | | | | | Awash Fentale | | | | 22 | | | | 28 | 0 | | 0 | | 50 | | | 32.5 | | |
|  | | | | | | | Amibara | | | | 0 | | | | 2 | 57 | | 35 | | 94 | | | 61.0 | | |
| No | | | | | | | Awash Fentale | | | | 0 | | | | 0 | 0 | | 0 | | 0 | | | 0.0 | | |
|  | | | | | | | Amibara | | | | 0 | | | | 0 | 0 | | 2 | | 2 | | | 1.3 | | |
| Neutral | | | | | | | Awash Fentale | | | | 0 | | | | 1 | 0 | | 0 | | 1 | | | 0.6 | | |
|  | | | | | | | Amibara | | | | 0 | | | | 0 | 1 | | 3 | | 4 | | | 2.6 | | |
| General view bout *P.juliflora*? | | | | | | | | | | |  | | | |  |  | |  | |  | | |  | | |
| grass land affected by *P.juliflora* | | | | | Awash Fentale | | | | | | 8 | | | | 5 | 0 | | 0 | | 13 | | | 8.4 | | |
|  | | | | | Amibara | | | | | | 0 | | | | 0 | 19 | | 3 | | 22 | | | 14.3 | | |
| farm land affected by *P.juliflora* | | | | | Awash Fentale | | | | | | 10 | | | | 0 | 0 | | 0 | | 10 | | | 6.5 | | |
|  | | | | | Amibara | | | | | | 0 | | | | 0 | 0 | | 0 | | 0 | | | 0.0 | | |
| Severe invasion LULC in the region | | | | | Awash Fentale | | | | | | 3 | | | | 1 | 0 | | 0 | | 4 | | | 2.6 | | |
|  | | | | | Amibara | | | | | | 0 | | | | 0 | 7 | | 6 | | 13 | | | 8.4 | | |
| Expansion of *P.juliflora* has induced poverty | | | | | Awash Fentale | | | | | | 1 | | | | 0 | 0 | | 0 | | 1 | | | 0.6 | |  |
|  | | | | | Amibara | | | | | | 0 | | | | 0 | 0 | | 0 | | 0 | | | 0.0 | |  |
| Toxic effects of *P.juliflora* | | | | | Awash Fentale | | | | | | 0 | | | | 0 | 0 | | 0 | | 0 | | | 0.0 | |  |
|  | | | | | Amibara | | | | | | 0 | | | | 0 | 2 | | 0 | | 2 | | | 1.3 | |  |
| It should be controlled | | | | | Awash Fentale | | | | | | 0 | | | | 23 | 0 | | 0 | | 23 | | | 14.9 | |  |
|  | | | | | Amibara | | | | | | 0 | | | | 2 | 30 | | 28 | | 60 | | | 39.0 | |  |
| Expansion of *P.juliflora* has affected livelihood of the region | | | | | Awash Fentale | | | | | | 0 | | | | 0 | 0 | | 0 | | 0 | | | 0.0 | |  |
|  | | | | | Amibara | | | | | | 0 | | | | 0 | 0 | | 3 | | 3 | | | 1.9 | |  |

## Appendix 14. Perceptions of key informants towards *P.juliflora* in South Afar Region

| Response variables | |  |  |  | |  |
| --- | --- | --- | --- | --- | --- | --- |
| When did *P.juliflora* come first? | | District | Frequency | | | % |
| 23 | | Awash Fentale | 4 | 4 | | 44.4 |
|  | | Amibara | 3 | 3 | | 33.3 |
| 28 | | Awash Fentale | 1 | 1 | | 11.1 |
|  | | Amibara | 0 | 0 | | 0.0 |
| 31 | | Awash Fentale | 0 | 0 | | 0.0 |
|  | | Amibara | 1 | 1 | | 11.1 |
| Do you know how *P.juliflora* introduced in your district? | | | | | | |
| Yes | Awash Fentale | | 3 | | 3 | 33.3 |
|  | Amibara | | 4 | | 4 | 44.4 |
| No | Awash Fentale | | 2 | | 2 | 22.2 |
|  | Amibara | | 0 | | 0 | 0.0 |
| if yes, how was *P.juliflora* introduced? | | | | | | |
| Intentional | | Awash Fentale | 4 | | 4 | 44.4 |
|  | | Amibara | 1 | | 1 | 11.1 |
| Natural dispersal | | Awash Fentale | 1 | | 1 | 11.1 |
|  | | Amibara | 2 | | 2 | 22.2 |
| Livestock | | Awash Fentale | 0 | | 0 | 0.0 |
|  | | Amibara | 1 | | 1 | 11.1 |
| If the introduction is intentional who brought *P.juliflora*? | | | | | | |
| GOV | | Awash Fentale | 0 | | 0 | 0.0 |
|  | | Amibara | 1 | | 1 | 11.1 |
| NGO's | | Awash Fentale | 2 | | 2 | 22.2 |
|  | | Amibara | 3 | | 3 | 33.3 |
| Individual persons | | Awash Fentale | 3 | | 3 | 33.3 |
|  | | Amibara | 0 | | 0 | 0.0 |
| Why *P.juliflora* introduced in your district? | | | | | | |
| Fuel wood purposes | | Awash Fentale | 2 | | 2 | 22.2 |
|  | | Amibara | 1 | | 1 | 11.1 |
| Shade purposes | | Awash Fentale | 2 | | 2 | 22.2 |
|  | | Amibara | 3 | | 3 | 33.3 |
| Soil and water conservation | | Awash Fentale | 1 | | 1 | 11.1 |
|  | | Amibara | 0 | | 0 | 0.0 |
| What is the preferred site for establishment of *P.juliflora*? | | | | | | |
| Homestead | | Awash Fentale | 1 | | 1 | 11.1 |
|  | | Amibara | 0 | | 0 | 0.0 |
| Wetlands | | Awash Fentale | 1 | | 1 | 11.1 |
|  | | Amibara | 0 | | 0 | 0.0 |
| Range lands | | Awash Fentale | 0 | | 0 | 0.0 |
|  | | Amibara | 3 | | 3 | 33.3 |
| Along rivers | | Awash Fentale | 3 | | 3 | 33.3 |
|  | | Amibara | 1 | | 1 | 11.1 |
| What is the topographic preference for the growth of *P.juliflora*? | | | | | | |
| Flat land | | Awash Fentale | 4 | | 4 | 44.4 |
|  | | Amibara | 3 | | 3 | 33.3 |
| Rivers | | Awash Fentale | 1 | | 1 | 11.1 |
|  | | Amibara | 1 | | 1 | 11.1 |
| How *P.juliflora* dispersed? | | | | | | |
| Cattle | | Awash Fentale | 2 | | 2 | 22.2 |
|  | | Amibara | 3 | | 3 | 33.3 |
| Goats | | Awash Fentale | 3 | | 3 | 33.3 |
|  | | Amibara | 0 | | 0 | 0.0 |
| Sheep | | Awash Fentale | 0 | | 0 | 0.0 |
|  | | Amibara | 1 | | 1 | 11.1 |
| What are major regeneration methods of *P.juliflora*? | | | | | | |
| Seeds | | Awash Fentale | 5 | | 5 | 55.6 |
|  | | Amibara | 1 | | 1 | 11.1 |
| Coppicing from cut stems | | Awash Fentale | 0 | | 0 | 0.0 |
|  | | Amibara | 3 | | 3 | 33.3 |
| What kinds of impacts of *P.juliflora* on biodiversity? | | | | |  |  |
| Positive | | Awash Fentale | 1 | | 1 | 11.1 |
|  | | Amibara | 2 | | 2 | 22.2 |
| Negative | | Awash Fentale | 4 | | 4 | 44.4 |
|  | | Amibara | 2 | | 2 | 22.2 |
| What type of biodiversity affected by *P.juliflora*? | | | | | | |
| Livestock | | Awash Fentale | 2 | | 2 | 22.2 |
|  | | Amibara | 4 | | 4 | 44.4 |
| Human beings | | Awash Fentale | 1 | | 1 | 11.1 |
|  | | Amibara | 0 | | 0 | 0.0 |
| Plants | | Awash Fentale | 2 | | 2 | 22.2 |
|  | | Amibara | 0 | | 0 | 0.0 |
| What type of biodiversity most affected by *P.juliflora*? | | | | | | |
| Livestock | | Awash Fentale | 3 | | 3 | 33.3 |
|  | | Amibara | 4 | | 4 | 44.4 |
| Plants | | Awash Fentale | 2 | | 2 | 22.2 |
|  | | Amibara | 1 | | 1 | 11.1 |
| Which effect of *P.juliflora* is higher in your district? | | | | | | |
| Negative | | 1 | 5 | | 5 | 55.6 |
|  | | 2 | 4 | | 4 | 44.4 |
| If yes, which wealth class uses *P.juliflora*? | | | | | | |
| Poor | | Awash Fentale | 4 | | 4 | 44.4 |
|  | | Amibara | 4 | | 4 | 44.4 |
| Medium | | Awash Fentale | 1 | | 1 | 11.1 |
|  | | Amibara | 0 | | 0 | 0.0 |
| What impacts of *P.juliflora* does on livestock in your district? | | | | | | |
| Injure livestock with poisonous thorns | | Awash Fentale | 0 | | 0 | 0.0 |
|  | | Amibara | 1 | | 1 | 11.1 |
| Change goats/sheep teeth to rot | | Awash Fentale | 0 | | 0 | 0.0 |
|  | | Amibara | 1 | | 1 | 11.1 |
| Change quality of milk | | Awash Fentale | 2 | | 2 | 22.2 |
|  | | Amibara | 1 | | 1 | 11.1 |
| Change quantity of milk | | Awash Fentale | 3 | | 3 | 33.3 |
|  | | Amibara | 1 | | 1 | 11.1 |
| What impacts does on specifically on plants? | | | | | | |
| Reduce plant diversity | | Awash Fentale | 2 | | 2 | 22.2 |
|  | | Amibara | 1 | | 1 | 11.1 |
| Toxic effects on plant species | | Awash Fentale | 1 | | 1 | 11.1 |
|  | | Amibara | 2 | | 2 | 22.2 |
| Overtake the site and inhibit other plant species not grow | | Awash Fentale | 1 | | 1 | 11.1 |
|  | | Amibara | 0 | | 0 | 0.0 |
| Share moisture with other naïve plants | | Awash Fentale | 1 | | 1 | 11.1 |
|  | | Amibara | 0 | | 0 | 0.0 |
| Others | | Awash Fentale | 1 | | 1 | 11.1 |
|  | | Amibara | 0 | | 0 | 0.0 |
| What impacts does on specifically on human beings in your site? | | | | | | |
| Long thorns are piercing and become toxic | | Awash Fentale | 2 | | 2 | 22.2 |
|  | | Amibara | 4 | | 4 | 44.4 |
| Block pass way | | Awash Fentale | 3 | | 3 | 33.3 |
|  | | Amibara | 3 | | 3 | 33.3 |
| What do you think is the Afar people's attitude towards *P.juliflora*? | | | | | | |
| It is as deleterious species | | Awash Fentale | 5 | | 5 | 55.6 |
|  | | Amibara | 4 | | 4 | 44.4 |
| Is the spread of *P.juliflora* increasing from time to time? | | | | | | |
| Yes | | Awash Fentale | 5 | | 5 | 55.6 |
|  | | Amibara | 4 | | 4 | 44.4 |
| What are the changes are you observing in livestock holdings? | | | | | | |
| Declining | | Awash Fentale | 4 | | 4 | 44.4 |
|  | | Amibara | 4 | | 4 | 44.4 |
| Increasing | | Awash Fentale | 1 | | 1 | 11.1 |
|  | | Amibara | 0 | | 0 | 0.0 |
| What are the changes? | | | | | | |
| Shortage of rainfall | | Awash Fentale | 2 | | 2 | 22.2 |
|  | | Amibara | 1 | | 1 | 11.1 |
| Agricultural expansion | | Awash Fentale | 0 | | 0 | 0.0 |
|  | | Amibara | 1 | | 1 | 11.1 |
| Invasion of *P.juliflora* | | Awash Fentale | 3 | | 3 | 33.3 |
|  | | Amibara | 2 | | 2 | 22.2 |
| How are the changing events responsible? | | | | | | |
| In terms of grazing land encroachment | | Awash Fentale | 1 | | 1 | 11.1 |
|  | | Amibara | 2 | | 2 | 22.2 |
| In terms declining of livestock production and productivity | | Awash Fentale | 3 | | 3 | 33.3 |
|  | | Amibara | 2 | | 2 | 22.2 |
| Others | | Awash Fentale | 1 | | 1 | 11.1 |
|  | | Amibara | 0 | | 0 | 0.0 |
| How is the grazing area availability after *P.juliflora* introduction? | | | | | | |
| Declining | | Awash Fentale | 4 | | 4 | 44.4 |
|  | | Amibara | 3 | | 3 | 33.3 |
| Increasing | | Awash Fentale | 1 | | 1 | 11.1 |
|  | | Amibara | 1 | | 1 | 11.1 |
| Which lULC converted last 10 years? | | | | | | |
| Acacia woodland to *P.juliflora* | | Awash Fentale | 1 | | 1 | 11.1 |
|  | | Amibara | 2 | | 2 | 22.2 |
| Acacia woodland to settlement | | Awash Fentale | 1 | | 1 | 11.1 |
|  | | Amibara | 0 | | 0 | 0.0 |
| Grass land to *P.juliflora* | | Awash Fentale | 2 | | 2 | 22.2 |
|  | | Amibara | 1 | | 1 | 11.1 |
| *P.juliflora* with other tree species | | Awash Fentale | 0 | | 0 | 0.0 |
|  | | Amibara | 1 | | 1 | 11.1 |
| Water body to *P.juliflora* | | Awash Fentale | 1 | | 1 | 11.1 |
|  | | Amibara | 0 | | 0 | 0.0 |
| Have you ever tried to control? | | | | | | |
| Yes | | Awash Fentale | 2 | | 2 | 22.2 |
|  | | Amibara | 4 | | 4 | 44.4 |
| No | | Awash Fentale | 3 | | 3 | 33.3 |
|  | | Amibara | 0 | | 0 | 0.0 |
| Which measure should be taken to control the spread of *P.juliflora?* | | | | | | |
| Manage by utilization | | Awash Fentale | 0 | | 0 | 0.0 |
|  | | Amibara | 1 | | 1 | 11.1 |
| Mechanical control by cutting mature stems | | Awash Fentale | 1 | | 1 | 11.1 |
|  | | Amibara | 1 | | 1 | 11.1 |
| Fire | | Awash Fentale | 1 | | 1 | 11.1 |
|  | | Amibara | 2 | | 2 | 22.2 |
| Chemicals | | Awash Fentale | 2 | | 2 | 22.2 |
|  | | Amibara | 0 | | 0 | 0.0 |
| Control seed dispersal | | Awash Fentale | 1 | | 1 | 11.1 |
|  | | Amibara | 0 | | 0 | 0.0 |
| Should *P.juliflora* eradicate from Afar region completely? | | | | | | |
| Yes | | Awash Fentale | 3 | | 3 | 33.3 |
|  | | Amibara | 4 | | 4 | 44.4 |
| Neutral | | Awash Fentale | 2 | | 2 | 22.2 |
|  | | Amibara | 0 | | 0 | 0.0 |
| If you agree, what will be done to control *P.juliflora* expansion? | | | | | | |
| Manage by utilization | | 1 | 5 | | 5 | 55.6 |
|  | | 2 | 4 | | 4 | 44.4 |
| What are the general ideas about the invasion of *P.juliflora*? | | | | | | |
| grazing land is overtaken and should be controlled | | Awash Fentale | 2 | | 2 | 22.2 |
|  | | Amibara | 4 | | 4 | 44.4 |
| Settlement areas overtaken and need control | | Awash Fentale | 3 | | 3 | 33.3 |
|  | | Amibara | 0 | | 0 | 0.0 |

## Appendix 15. Effects of districts on perceptions of communities towards introduction, phenology, use and effects of *P.juliflora* by in South Afar Region

| Response variables | | | | | | | | | | | | | | | | | | | | |  | | | |  | | |  |  |  |
| --- | --- | --- | --- | --- | --- | --- | --- | --- | --- | --- | --- | --- | --- | --- | --- | --- | --- | --- | --- | --- | --- | --- | --- | --- | --- | --- | --- | --- | --- | --- |
| Stat | | MOL | | DHp | IYH | | IIPWI | | | WIn | | | | PSreg | | | | BPJ | | | IPoNr | | | | DCmU | | |  |  |  |
| *χ^2^* | | 37.99 | | 0.05 | 6.62 | | 0.98 | | | 12.38 | | | | 14.34 | | | | 18.74 | | | 0.67 | | | | 0.00 | | |  |  |  |
| df | | 1 | | 1 | 1 | | 1 | | | 1 | | | | 1 | | | | 1 | | | 1 | | | | 1 | | |  |  |  |
| *P-value* | | <0.0001 | | 0.82 | 0.01 | | 0.32 | | | <0.0001 | | | | <0.0001 | | | | <0.0001 | | | 0.41 | | | | 0.99 | | |  |  |  |
| Stat | | Wcl | DPUH | | PPu | | PHF | | DYPTr | | | | prepTr | | | | HDise | | | LivDise | | | | PrLiveM | | |  | |  |  |
| *χ^2^* | | 1.04 | 0.98 | | 0.78 | | 1.82 | | 9.03 | | | | 7.62 | | | | 8.55 | | | 6.77 | | | | 8.32 | | |  | |  |  |
| df | | 1 | 1 | | 1 | | 1 | | 1 | | | | 1 | | | | 1 | | | 1 | | | | 1 | | |  | |  |  |
| *P-value* | | 0.31 | 0.32 | | 0.38 | | 0.18 | | 0.003 | | | | 0.006 | | | | 0.003 | | | 0.01 | | | | 0.004 | | |  | |  |  |
| **Notices:** Stat is Statistics, MOL is Mode of living, DHp is do you know how *P.juliflora* introduced, IYH is If yes how was introduced, IIPWI is If *P.juliflora* introduced was intentional, who brought, WIn is Why was *P.juliflora* introduced in your site?, PSreg Preferred site for *P.juliflora* regeneration, BPJ is What benefits you get from *P.juliflora?* IPoNr is which do you think positive or negative impacts of *P.juliflora* higher? DCmU is do communities use *P.juliflora* in your district?, Wcl is Which wealth class mostly uses the *P.juliflora*?, DPUH is does *P.juliflora* use for human food? PPu is Which part of *P.juliflora* use for human food?, PHF is List preparation of human food from *P.juliflora,* DYPTr is Do you think *P.juliflora* is used for traditional medicine? prepTr is Preparation of *P.julifolra* for traditional medicine?, HDis is human disease, LivDis is Livestock disease, PrLiveM is Preparation method for traditional medicine for livestock diseases from *P.juliflora?* Appendix 16. Perceptions of districts on communities towards the effects of *P.juliflora* in South Afar Region Response variables | | | | | | | | | | | | | | | | | | | | | | | | | | | |  |  |  |
| Stat | IPJ | | NIPjLS | | | NIPJPl | | WIpjpl | | | DOIBD | | | | WKBDA | | | | WTBDA | | | MABDPjI | | | | IPJEnc | |  |  |  |
| *χ^2^* | 6.80 | | 0.23 | | | 8.21 | | 0.19 | | | 7.68 | | | | 3.38 | | | | 1.02 | | | 3.55 | | | | 2.26 | |  |  |  |
| df | 1 | | 1 | | | 1 | | 1 | | | 1 | | | | 1 | | | | 1 | | | 1 | | | | 1 | |  |  |  |
| *P-value* | 0.01 | | 0.64 | | | 0.004 | | 0.66 | | | 0.01 | | | | 0.07 | | | | 0.31 | | | 0.06 | | | | 0.13 | |  |  |  |
| Stat | | DPJIPl | | PECat | | PESh | | PEGt | | | | PECm | | | | PEDo | | | DOCon | | | | IyWDAAf | | |  | |  |  |  |
| *χ^2^* | | 4.42 | | 3.97 | | 5.04 | | 4.63 | | | | 10.71 | | | | 2.62 | | | 1.55 | | | | 7.94 | | |  | |  |  |  |
| df | | 1 | | 1 | | 1 | | 1 | | | | 1 | | | | 1 | | | 1 | | | | 1 | | |  | |  |  |  |
| *P-value* | | 0.04 | | 0.05 | | 0.03 | | 0.03 | | | | 0.001 | | | | 0.12 | | | 0.21 | | | | 0.01 | | |  | |  |  |  |

Notices: Stat is Statistics, IPJ is What are impacts of *P.juliflora?,* NIPJLS is Negative effects of *P.juliflora* on livestock?, NIPjPl is Negative effects of *P.juliflora* on plants?, WIpjpl is Negative effects of *P.juliflora* on plants?, DOIBD is Do think *P.juliflora* will have impacts on biodiversity?, WKBDA is What kinds of impacts on biodiversity?, WTBDA is What type of biodiversity affected by *P.juliflora*?, MABDPjI is most affected biodiversity, IPJEnc is How is *P.juliflora* encroachment influencing livestock productivity in your site?, DPJIPl is Do *P.juliflora* impacts on plants?, PECat is Which part of *P.juliflora* is eaten by cattle?, PESh is Which part of *P.juliflora* is eaten by sheep?, PEGt is Which part of *P.juliflora* is eaten by goats?, PECm is PEcWhich part of *P.juliflora* is eaten by camels?, PEDo is Which part of *P.juliflora* is eaten by donkeys?, , DOCon is Did you observed the concentration of *P.juliflora* in taking of animals?, IyWDAAF is If yes, which domestic animal most affected by *P.juliflora?*

## Appendix 17. Perceptions of districts on communities towards phenology, encroachment effects, and control measures of *P.juliflora* in South Afar Region

| Response variables | | | | | | | | | | | | | | | | | | | | | | | |  |
| --- | --- | --- | --- | --- | --- | --- | --- | --- | --- | --- | --- | --- | --- | --- | --- | --- | --- | --- | --- | --- | --- | --- | --- | --- |
| Stat | | HPD | | GPJ | | FMPJ | | SSMPJ | | | LMDPJ | | WADPJ | | | | MAxSSP | | MPJRM | | DCPCop | | IYNo |  |
| *χ^2^* | | 0.13 | | 3.78 | | 0.81 | | 0.23 | | | 11.91 | | 0.04 | | | | 1.62 | | 0.42 | | 28.18 | | 2.20 |  |
| df | | 1 | | 1 | | 1 | | 1 | | | 1 | | 1 | | | | 1 | | 1 | | 1 | | 1 |  |
| *P-value* | | 0.72 | | 0.05 | | 0.37 | | 0.63 | | | 0.001 | | 0.84 | | | | 0.20 | | 0.52 | | <0.000 | | 0.12 |  |
| Stat | SPINCde | | CCh | | | Hchere | LUC10 | | | CLULCC | | | | CantrC | | Mcontrl | | | DUPtE WDCPJ | | |  |  |  |
| *χ^2^* | 0.14 | | 2.65 | | | 3.31 | 0.32 | | | 2.00 | | | | 1.97 | | 10.03 | | | 0.62 6.68 | | |  | |  |
| df | 1 | | 1 | | | 1 | 1 | | | 1 | | | | 1 | | 1 | | | 1 1 | | |  | |  |
| *P-value* | 0.72 | | 0.10 | | | 0.07 | 0.58 | | | 0.16 | | | | 0.16 | | 0.002 | | | 0.43 0.01 | | |  | |  |
| Notices: Stat is statistics, *P<0.05,* df is degree of freedom, HPD is How do think *P.juliflora* dispersed?, GPJ is Lush growth of *P.juliflora?,* FMPJ is Flower month, SSMPJ is Seed set of *P.juliflora*?, LMDPJ is Livestock *P.juliflora*.j most dispersed?, WADPJ is Wild animal most dispersed *P.juliflora?,* MAxSSP is Month of maximum seed set/dispersal for *P.juliflora?,* MPJRM is Major *P.juliflora* regeneration?, DCPCop is Did you count *P.juliflora* coppices?, IYNo is If yes number of coppices?, SPINCde is Is spread of *P.juliflora* increasing or declining?, CCh is How the changes above are responsible?, Hchere is How grazing land availability after introduction of *P.juliflora*?, LUC10 is Which LULC converted last 10 years?, CLULCC is What are the causes for LULC conversions?, CantrC is Have you ever tried to control invasion of *P.juliflora*?, Mcontrl is Which measures are taken to control *P.juliflora*?, DUPtE Is *P.juliflora* eradicated from Afar region?, WDCPJ is if you agree to eradicate *P.juliflora*, what should be done? Appendix 18. Perceptions of districts on communities towards dwelling of wild life under *P.juliflora* in South Afar Region Response variables | | | | | | | | | | | | | | | | | | | | | | | |  |
| Stat | | AreWLU | | | ArePGUPJ | | IFYWpl | | DDAP | | | ISPJ | | | NEPFCr | | | NEPJR | | WWPPJKA | | | WPPJIH |  |
| *χ^2^* | | 9.91 | | | 8.81 | | 3.74 | | 17.45 | | | 0.23 | | | 2.03 | | | 6.77 | | 10.36 | | | 0.13 |  |
| df | | 1 | | | 1 | | 1 | | 1 | | | 1 | | | 1 | | | 1 | | 1 | | | 1 |  |
| *P-value* | | 0.002 | | | 0.003 | | 0.05 | | <0.0001 | | | 0.629 | | | 0.154 | | | 0.009 | | 0.001 | | | 0.721 |  |

Notices: Stat is statistics, *P < 0.05,* df is degree of freedom, AreWLU is Are wild lives under *P.juliflora*? ArePGUPJ = are plants grow under *P.juliflora*?, IFYWpl if yes which plant?, DDAP is Do your domestic animals prefer the tree?, ISPJ is In which season do the animals graze/browse on the species? NEPFCr is Disadvantage/negative effects does *P.juliflora* have for your crops, NEPJR is Disadvantage/negative effects does *P.juliflora* have for the rangeland; WWPPJKA is Parts of *P.juliflora* kill animals? WPPJIH is what impacts does *P.juliflora* specifically on human beings in your site?

## Appendix 19. Effects of site on perceptions of communities towards introduction, phenology, use and effects of *P.juliflora* by in South Afar Region

| Response variables | | | | | | | | | |  | |  |  |
| --- | --- | --- | --- | --- | --- | --- | --- | --- | --- | --- | --- | --- | --- |
| Stat | MOL | DHpJint | | IYH | IIPWI | PSreg | TopoprePj | LushPJ | | FlowerM | |  |  |
| *χ^2^* | 55.59 | 8.64 | | 17.07 | 15.16 | 31.98 | 21.95 | 14.75 | | 16.77 | |  |  |
| df | 3 | 3 | | 3 | 3 | 3 | 3 | 3 | | 3 | |  |  |
| *P-value* | <0.0001 | 0.03 | | 0.001 | 0.002 | <0.0001 | <0.0001 | 0.003 | | 0.001 | |  |  |
| Stat | SePJ | LivMd | WldMd | | MMset | MPJreg | DCCop | MMliv | WBPJ | | DPJHfod | |  |
| *χ^2^* | 22.57 | 16.78 | 16.06 | | 13.44 | 14.3 | 27.24 | 9.95 | 22.36 | | 13.23 | |  |
| df | 3 | 3 | 3 | | 3 | 3 | 3 | 3 | 3 | | 3 | |  |
| *P-value* | <0.0001 | 0.001 | 0.001 | | 0.004 | 0.003 | <0.0001 | 0.02 | <0.0001 | | 0.004 | |  |
| *Stat* | WpartH | ListPPjH | DPJMd | | PreMd | LitHDis | LiLivDis | PremedP | WImPj | | NegIJPP | |  |
| *χ2* | 11.78 | 10.48 | 17.73 | | 13.65 | 14.81 | 10.93 | 11.15 | 13.39 | | 14.76 | |  |
| *df* | 3 | 3 | 3 | | 3 | 3 | 3 | 3 | 3 | | 3 | |  |
| *P-value* | 0.008 | 0.02 | 0.0005 | | 0.003 | 0.002 | 0.01 | 0.01 | 0.004 | | 0.002 | |  |
| *Stat* | DPJIBD | BDMAf | MAfBD | | PJEliE | DisPJR | WpPJKA | HCRRes | WLUPJ | | APGUPJ | |  |
| *χ2* | 12.04 | 8.41 | 8.21 | | 14.83 | 14.71 | 26.20 | 10.62 | 19.36 | | 21.45 | |  |
| *df* | 3 | 3 | 3 | | 3 | 3 | 3 | 3 | 3 | | 3 | |  |
| *P-value* | 0.007 | 0.04 | 0.042 | | 0.002 | 0.002 | <0.0001 | 0.01 | 0.0002 | | <0.0001 | |  |
| *Stat* | IYWpG | DaPPJ | WpPJCat | | WPJca | MoaAf | IYaMoA | HEcont | WMCor | | GenOv | |  |
| *χ2* | 21.45 | 31.45 | 9.56 | | 13.54 | 16.45 | 10.48 | 10.48 | 24.23 | | 10.47 | |  |
| *df* | 3 | 3 | 3 | | 3 | 3 | 3 | 3 | 3 | | 3 | |  |
| *P-value* | <0.0001 | <0.0001 | 0.02 | | 0.004 | 0.001 | 0.02 | 0.02 | <0.0001 | | 0.02 | |  |

Notices: Stat is statistics, MOL is mode of living, DHpJint is do you know how *P.juliflora* introduced? IYH is If yes how was *P.juliflora* introduced? IIPWI is If yes, was introduction of *P.juliflora* intentionally?, PSreg is preference site for *P.juliflora* establishment? , TopoprePj suitable topography for *P.juliflora* establishment, LushPJ is month of *P.juliflora,* FlowerM is flower month of *P.juliflora,* SePJ is month of seed set for *P.juliflora,* LivMd is livestock most dispersed *P.juliflora* seeds, WldMd is wildlife most dispersed *P.juliflora* seeds, MMset is month of maximum seed set *P.juliflora*?, MPJreg is major regeneration method of *P.juliflora*, DCCop is did counted coppices of *P.juliflora*, MMliv is most mode of living, WBPJ is what benefits you get from *P.juliflora*, DPJHfod is do *P.juliflora* use for human food?, WpartH is which part of *P.juliflora* used for human food, ListPPjH is list of preparation of human food from *P.juliflora*, DPJMd is do *P.juliflora* use for traditional medicine, PreMd is preparation traditional medicine, LitHDis is list of human diseases, LiLivDis list of livestock disease list, PremedP is preparation of traditional medicine for livestock, WImPj is what impacts do *P.juiflora*?, NegIJPP is negative impacts of *P.juliflora* on plants, DPJIB is do you think the impacts of *P.juliflora* on biodiversity, BDMAf is kind of biodiversity affected by *P.juliflora*, MAfBD is most affected biodiversity, PJEliE is *P.juliflora* effects encroachment on livestock productivity, DisPJR is disadvantage of *P.juliflora* on rangeland?, WpPJKA is which part of *P.juliflora* killing animals?, HCRRes is what are the causes for changes?, WLUPJ is are wildlife dwelling under *P.juliflora?,*  APGUPJ is are plants grow under *P.juliflora*, IYWpG is if yes, which plants grow under *P.juliflora*, DaPPJ is do animals prefer *P.juliflora* for conception, WpPJCa is which part of *P.juliflora* eaten by cattle, WPJca is which part of *P.juliflora* eaten by camels, MoaAf is most animals affected consumption of *P.juliflora*, IYaMoA is if yes, which animals affected, HEcont is have you ever tried to control *P.juliflora*?, WMCor is which measures are used to control *P.juliflora*?, GenOv is general view about *P.juliflora.*

## Appendix 20. Effects of education of household members on perceptions of communities towards *P.juliflora* in South Afar Region

| Stat | MOL | WInPJ | PreSPJ | MoLiv | DCcop |  |
| --- | --- | --- | --- | --- | --- | --- |
| *χ^2^* | 12.0 | 8.50 | 8.10 | 10.90 | 12.10 |  |
| df | 3 | 3 | 3 | 3 | 3 |  |
| *P-value* | 0.007 | 0.04 | 0.04 | 0.01 | 0.007 |  |

Notices: MOL is mode of living, WInPJ is what type of biodiversity is affected by *P.juliflora,* MoLiv is most mode living, DCcop is did counted coppiced of *P.juliflora*.

## Appendix 21. Effects of education of household head on perceptions of communities towards *P.juliflora* in South Afar Region

| Stat | MOL | WTBDAf | InWSnaCPJ | WpPJEa |  |
| --- | --- | --- | --- | --- | --- |
| *χ^2^* | 8.60 | 10.5 | 8.9 | 9.2 |  |
| df | 3 | 3 | 3 | 3 |  |
| *P-value* | 0.04 | 0.02 | 0.03 | 0.03 |  |

Notices: MOL is mode of living, WTBDAf is what type of biodiversity is affected by *P.juliflora,* InWSnaCPJ is in which season animals consume *P.juliflora*, WpPJEa is which part of *P.juliflora* is palatable by animals?

## Appendix 22. Effects of education of household type on perceptions of communities towards *P.juliflora* in South Afar Region

| Stat | MOL | PreSPJ | TopoPrePJ | HDPJdisper | Wadisper | NegePJLiv | Iinincodec |
| --- | --- | --- | --- | --- | --- | --- | --- |
| *χ^2^* | 23.9 | 12.97 | 13.19 | 15.31 | 24.19 | 15.49 | 14.67 |
| df | 6 | 6 | 6 | 6 | 6 | 6 | 6 |
| *P-value* | 0.001 | 0.04 | 0.04 | 0.02 | 0.0005 | 0.02 | 0.02 |
| *Stat* | WpEdonk | WPPJD | WMContPJ | HDPJdisper |  |  |  |
| *χ2* | 23.9 | 15.05 | 16.06 | 15.31 |  |  |  |
| *df* | 6 | 6 | 6 | 6 |  |  |  |
| *P-value* | 0.001 | 0.02 | 0.01 | 0.02 |  |  |  |

Notices: MOL is mode of living, PreSPJ is preferred site *for P.juliflora* establishment*,* TopoPrePJ is topography preferred for *P.juliflora* establishment, HDPJdisper is *P.juliflora* how does *P.julifora* dispersed seed, Wadisper is wild animal most dispersed seeds of *P.juliflora*, NegePJLiv is negative effects of *P.juliflora* on livestock, Iinincodec is the invasion of *P.juliflora* increasing/declining?, WpEdonk is which part of P.juliflora eaten by donkeys?, WPPJD what is the dispersing agent of *P.juliflora*, WMContPJ is what measure taken to control *P.juliflora*, HDPJdisper is how does *P.juliflora* seeds dispersed?

## Appendix 23. Effects whether household have position/not in kebele on perceptions of communities towards *P.juliflora* in South Afar Region

| Do have position in your site? | | | | Position and perception of *P.juliflora* invasion | | | |
| --- | --- | --- | --- | --- | --- | --- | --- |
| Stat | WWPJIn | PrefSPJ | WBPJ | IYnCopp | MoMLiv | DisaPJ |  |
| *χ^2^* | 5.44 | 5.50 | 9.64 | 20.49 | 21.2 | 10..93 |  |
| df | 1 | 1 | 1 | 1 | 1 | 1 |  |
| *P-value* | 0.02 | 0.02 | 0.002 | 0.0004 | 0.0003 | 0.03 |  |

**Notices:** WWPJIn is why was *P.juliflora* introduced?, PrefSPJ is preferred site for *P.juliflora*, WBPJ is what benefits do you get from *P.juliflora*, IYnCopp is if yes, number of coppices of *P.juliflora*, MoMLiv is most modeof living, DisaPJ is disadvantage of *P.juliflora*.

## Appendix 24. Effects of household relationships on perceptions of communities towards *P.juliflora* in South Afar Region

| Stat | IYnCopp | DaPrePJ |  |
| --- | --- | --- | --- |
| *χ^2^* | 15.12 | 10.78 |  |
| df | 4 | 4 |  |
| *P-value* | 0.004 | 0.03 |  |

Notices: IYnCopp is if yes, number of coppices of *P.juliflora,* DaPrePJ is do animals prefer *P.juliflora*.

## Appendix 25. Effects of sex of household on perceptions of communities towards *P.juliflora* in South Afar Region

| Stat | MoMaxsset | DPJHf | WPPJHf | WPPJKa | IyWPGUPJ | WPPJCat |  |
| --- | --- | --- | --- | --- | --- | --- | --- |
| *χ^2^* | 5.51 | 4.31 | 5.04 | 4.70 | 4.08 | 4.77 |  |
| df | 1 | 1 | 1 | 1 | 1 | 1 |  |
| *P-value* | 0.02 | 0.04 | 0.03 | 0.03 | 0.04 | 0.03 |  |

Notice: MoMaxsset is month of maximum seed set of *P.juliflora,* DPJHf does *P.juliflora* use for human food?, WPPJHf is which part of *P.juliflora* used for human food?, WPPJKa is which part of *P.juliflora* killing animals?, IyWPGUPJ is If yes, what types of plants grow under *P.juliflora*, WPPJCat is which part of *P.juliflora* is palatable by cattle?

## Appendix 26. Effects of wealth status of household on perceptions of communities towards *P.juliflora* in South Afar Region

| Stat | MOL | MostMOL | WWilMAfBD |  |
| --- | --- | --- | --- | --- |
| *χ^2^* | 6.75 | 8.98 | 9.17 |  |
| df | 2 | 2 | 2 |  |
| *P-value* | 0.03 | 0.01 | 0.01 |  |

**Notice:** MOL is mode of living MostMOL is most of living means, WWilMAfBD is which will be the most affected biodiversity.

## Appendix 27. Perception of communities by sex and wealth towards *P.juiflora* invasion effects in South Afar region

|  | SEX | | | | | | | |
| --- | --- | --- | --- | --- | --- | --- | --- | --- |
| Maximum month of *P.juliflor* seed set | | | | | | | | |
|  |  | | Male | | Female | | Total | |
|  | Month | | Number | % | Number | % | Number | % |
|  | September | | 6 | 3.90 | 7 | 4.55 | 13 | 8.44 |
|  | October | | 2 | 1.30 | 4 | 2.60 | 6 | 3.90 |
|  | November | | 5 | 3.25 | 3 | 1.95 | 8 | 5.19 |
|  | May | | 44 | 28.57 | 11 | 7.14 | 55 | 35.71 |
|  | June | | 8 | 5.19 | 2 | 1.30 | 10 | 6.49 |
|  | August | | 12 | 7.79 | 3 | 1.95 | 15 | 9.74 |
|  | March | | 1 | 0.65 | 1 | 0.65 | 2 | 1.30 |
|  | February | | 24 | 15.58 | 12 | 7.79 | 36 | 23.38 |
|  | December | | 3 | 1.95 | 2 | 1.30 | 5 | 3.25 |
|  | Throughout the year | | 1 | 0.65 | 1 | 0.65 | 2 | 1.30 |
|  | Total | | 106 | 68.83 | 46 | 29.87 | 152 | 98.70 |
|  | Does *P.juliflora* use for human food? | | | | | | | |
|  |  | | Male | | Female | | Total | |
|  |  | | Number | % | Number | % | Number | % |
|  | Yes | | 30 | 19.48 | 6 | 3.90 | 36 | 23.38 |
|  | No | | 73 | 47.40 | 39 | 25.32 | 112 | 72.73 |
|  | Total | | 103 | 66.88 | 45 | 29.22 | 148 | 96.10 |
|  | Which part of *P.juliflora* use for human food? | | | | | | | |
|  | Sex | | Male | | Female | | Total | |
|  | Plant parts | | Number | % | Number | % | Number | % |
|  | leaf | | 3 | 1.95 | 1 | 0.65 | 4 | 2.60 |
|  | seeds | | 100 | 64.94 | 44 | 28.57 | 144 | 93.51 |
|  | Total | | 103 | 66.88 | 45 | 29.22 | 148 | 96.10 |
|  | Which part of *P.juliflora* killing animals? | | | | | | | |
|  | Sex | | Male | | Female | | Total | |
|  | Plant part | | Number | % | Number | % | Number | % |
|  | leaf | | 101 | 65.58 | 45 | 29.22 | 4 | 2.60 |
|  | Thorn | | 2 | 1.30 | 0 | 0.00 | 2 | 1.30 |
|  | Pods/seeds | | 96 | 62.34 | 41 | 26.62 | 117 | 76.00 |
|  | Plant juice | | 3 | 1.95 | 2 | 1.30 | 5 | 3.25 |
|  | Total | | 103 | 66.88 | 45 | 29.22 | 148 | 96.10 |
| If yes, which plants grow under *P.juliflora*? | | | | | | | | |
| Sex | | Male |  | Female |  |  | Total |  |
|  | | Frequency | Number | % | Number | % | Number | % |
|  | | Perennial | 33 | 21.43 | 9 | 5.84 | 42 | 27.27 |
|  | | Annual | 4 | 2.60 | 0 | 0.00 | 4 | 2.60 |
|  | | Woody species | 66 | 42.86 | 36 | 23.38 | 102 | 66.23 |
|  | | Total | 103 | 66.88 | 45 | 29.22 | 148 | 96.10 |
|  | | Which part of *P.juliflora* palatable by animals? | | | | | | |
|  | | Sex | Male |  | Female | | Total | |
|  | | Respondents | Number | % | Number | % | Number | % |
|  | | leaf | 18 | 11.69 | 9 | 5.84 | 27 | 17.53 |
|  | | pod/seed | 81 | 52.60 | 34 | 22.08 | 115 | 74.68 |
|  | | bark | 2 | 1.30 | 0 | 0.00 | 2 | 1.30 |
|  | | flower | 2 | 1.30 | 2 | 1.30 | 4 | 2.60 |
|  | | Total | 103 | 66.88 | 45 | 29.22 | 148 | 96.10 |
|  | | Which part eaten *P.juliflora* by cattle? | | | |  |  |  |
|  | |  | Male |  | Female |  | Total |  |
|  | |  | Frequency | % | Frequency | % | Frequency | % |
|  | | leaf | 17 | 11.04 | 13 | 8.44 | 30 | 19.48 |
|  | | roots |  | 0 | 2 | 1.3 | 6 | 3.9 |
|  | | seed/pod | 80 | 51.95 | 27 | 17.53 | 107 | 69.48 |
|  | | flower | 2 | 1.3 | 3 | 1.95 | 5 | 3.25 |
|  | | Total | 103 | 66.88 | 45 | 29.22 | 148 | 96.1 |

WEALTH

| Which will be the most affected BD? | | | | | | | | |
| --- | --- | --- | --- | --- | --- | --- | --- | --- |
|  | Poor | | Medium | | Rich | | Total | |
|  | Frequency | % | Frequency | % | Frequency | % | Number % | |
| Livestock | 65 | 42.21 | 48 | 31.17 | 1 | 0.65 | 114 | 74.03 |
| Plant | 25 | 16.23 | 4 | 2.60 | 1 | 0.65 | 30 | 19.48 |
| Human beings | 1 | 0.65 | 0 | 0.00 | 0 | 0.00 | 1 | 0.65 |
| Wild animals | 0 | 0.00 | 2 | 1.30 | 1 | 0.65 | 3 | 1.95 |
| Total | 91 | 59.09 | 54 | 35.06 | 3 | 1.95 | 148 | 96.104 |

## Appendix 28. Perception of communities by educations of household, household types, and positions in their kebeles towards *P.juiflora* invasion effects in South Afar region

| Education of HOUSEHOLD member | | | | | | | | | | | | | | | | | | | | | |  |
| --- | --- | --- | --- | --- | --- | --- | --- | --- | --- | --- | --- | --- | --- | --- | --- | --- | --- | --- | --- | --- | --- | --- |
| If *P.juliflora* introduced, was intentional who brought? | | | | | | | | |  | | |  | |  | |  | |  | |  |  |  |
|  | No formal | | | | | | Primary | | Secondary | | | | | Post-secondary | | | | Total | |  |  |  |
|  | Number | | | | % | | Number | % | Number | | | | % | Number | | | % | Number | | % |  |  |
| GOV authorities | 11 | | | | 7.14 | | 12 | 7.79 | 6 | | | | 3.90 | 0 | | | 0.00 | 30 | | 19.48 |  |  |
| NGO's | 33 | | | | 21.43 | | 23 | 14.94 | 4 | | | | 2.60 | 7 | | | 4.55 | 67 | | 43.51 |  |  |
| Individual persons | 19 | | | | 12.34 | | 24 | 15.58 | 7 | | | | 4.55 | 4 | | | 2.60 | 55 | | 35.71 |  |  |
| Total | 63 | | | | 40.91 | | 59 | 38.31 | 17 | | | | 11.04 | 11 | | | 7.14 | 152 | | 98.70 |  |  |
|  |  | | | |  | |  |  |  | | | |  |  | | |  |  | |  |  |  |
| Preferred site for *P.juliflora*  establishment? | | | | | | | | |  | | | |  |  | | |  |  | |  |  |  |
|  | No formal | | | | | | Primary | | Secondary | | | | | Post-secondary | | | | Total | |  |  |  |
|  | Number | | | | % | | Number | % | Number | | | | % | Number | | | % | Number | | % |  |  |
| Homestead | 20 | | | | 12.99 | | 15 | 9.74 | 3 | | | | 1.95 | 1 | | | 0.65 | 39 | | 25.32 |  |  |
| Road sides | 9 | | | | 5.84 | | 8 | 5.19 | 0 | | | | 0.00 | 2 | | | 1.30 | 20 | | 12.99 |  |  |
| Wetlands | 0 | | | | 0.00 | | 7 | 4.55 | 1 | | | | 0.65 | 4 | | | 2.60 | 12 | | 7.79 |  |  |
| Rangelands | 23 | | | | 14.94 | | 21 | 13.64 | 6 | | | | 3.90 | 2 | | | 1.30 | 53 | | 34.42 |  |  |
| Around mechanized farms | 10 | | | | 6.49 | | 8 | 5.19 | 6 | | | | 3.90 | 2 | | | 1.30 | 26 | | 16.88 |  |  |
| Along rivers | 0 | | | | 0.00 | | 0 | 0.00 | 1 | | | | 0.65 | 0 | | | 0.00 | 1 | | 0.65 |  |  |
| woodlands | 1 | | | | 0.65 | | 0 | 0.00 | 0 | | | | 0.00 | 0 | | | 0.00 | 1 | | 0.65 |  |  |
| Total | 63 | | | | 40.91 | | 59 | 38.31 | 17 | | | | 11.04 | 11 | | | 7.14 | 152 | | 98.70 |  |  |
| Education of HOUSEHOLD MEMBER | | | | | | | | | | | | | | | | | | | | | | |
|  | Did you count *P.juliflora* coppices? | | | | | | | |  | | | |  |  | | |  |  | | | |  |
|  | Yes | | | |  | | No |  |  | | | |  |  | | |  |  | | | |  |
|  | Number | | | | % | | Number | % |  | | | |  |  | | |  |  | | | |  |
| No formal education | 7 | | | | 4.55 | | 86 | 55.84 |  | | | |  |  | | |  |  | | | |  |
| Primary | 20 | | | | 12.99 | | 41 | 26.62 |  | | | |  |  | | |  |  | | | |  |
| Secondary | 1 | | | | 0.65 | | 17 | 11.04 |  | | | |  |  | | |  |  | | | |  |
| Post-secondary | 2 | | | | 1.30 | | 10 | 6.49 |  | | | |  |  | | |  |  | | | |  |
| Total | 31 | | | | 20.13 | | 154 | 100.00 |  | | | |  |  | | |  |  | | | |  |
| Education of HOUSEHOLD head | | | | | | | | | | | | | | | | | | | | | | |
| Earn living modes? | | | | | | | | | | | | | | | | | | | | | |  |
|  | Pastoralists | | | | | | Petty trade | | Governmet employee | | | | | Total | | | |  | | | |  |
|  | Number | | | | % | | Number | % | Number | | | | % | Number | | | % |  | | | |  |
| No formal education | 95 | | | | 61.69 | | 3 | 1.948 | 8 | | | | 5.19 | 144 | | | 93.51 |  | | | |  |
| Primary | 2 | | | | 1.30 | | 0 | 1.948 | 0 | | | | 0.00 | 2 | | | 1.30 |  | | | |  |
| Secondary | 2 | | | | 1.30 | | 0 | 1.948 | 1 | | | | 0.65 | 4 | | | 2.60 |  | | | |  |
| Post-secondary | 1 | | | | 0.65 | | 0 | 1.948 | 1 | | | | 0.65 | 4 | | | 2.60 |  | | | |  |
| Total | 100 | | | | 64.94 | | 3 | 1.948 | 8 | | | | 5.19 | 154 | | | 100.00 |  | | | |  |
|  |  | | | |  | |  |  |  | | | |  |  | | |  |  | | | |  |
| What type of BD affected by *P.juliflora?* | | | | | | | | | | | | | | | | | | | | | |  |
|  | No formal | | | | | | Primary | | Total | | | | |  | | |  |  | | | |  |
|  | Number | | | | % | | Number | % | Number | | | | % |  | | |  |  | | | |  |
| Livestock | 106 | | | | 68.83 | | 2 | 1.30 | 109 | | | | 70.78 |  | | |  |  | | | |  |
| Plant | 32 | | | | 20.78 | | 0 | 0.00 | 37 | | | | 24.03 |  | | |  |  | | | |  |
| Human being | 2 | | | | 1.30 | | 0 | 0.00 | 2 | | | | 1.30 |  | | |  |  | | | |  |
| Total | 140 | | | | 90.91 | | 2 | 1.30 | 148 | | | | 96.10 |  | | |  |  | | | |  |
| In which season do animals feed on *P.juliflora?* | | | | | | | | | | | | | | | | | | | | | |  |
|  | No formal | | | | | | Primary | | Total | | | | |  | | |  |  | | | |  |
|  | Number | | | | % | | Number | % | Number | | | | % |  | | |  |  | | | |  |
| Dry | 132 | | | | 85.71 | | 1 | 0.65 | 133 | | | | 86.36 |  | | |  |  | | | |  |
| Wet | 8 | | | | 5.19 | | 1 | 0.65 | 9 | | | | 5.84 |  | | |  |  | | | |  |
| Total | 140 | | | | 90.91 | | 2 | 1.30 | 142 | | | | 92.21 |  | | |  |  | | | |  |
| Which part *P.juliflora* palatable by animals? | | | | | | | | | | | | | | | | | | | | | |  |
|  | No formal | | | | | | Primary | | Total | | | | |  | | |  |  | | | |  |
|  | Number | | | | % | | Number | % | Number | | | | % |  | | |  |  | | | |  |
| Leaf | 23 | | | | 14.94 | | 2 | 1.30 | 27 | | | | 17.53 |  | | |  |  | | | |  |
| Pod | 111 | | | | 72.08 | | 0 | 0.00 | 113 | | | | 73.38 |  | | |  |  | | | |  |
| Bark | 2 | | | | 1.30 | | 0 | 0.00 | 2 | | | | 1.30 |  | | |  |  | | | |  |
| Flower | 4 | | | | 2.60 | | 0 | 0.00 | 4 | | | | 2.60 |  | | |  |  | | | |  |
| Total | 140 | | | | 90.91 | | 2 | 1.30 | 148 | | | | 96.10 |  | | |  |  | | | |  |
| HOUSEHOLD types | | | | | | | | | | | | | | | | | | | | | |  |
| Earn living modes? | | | | | | | | | | | | | | | | | | | | | |  |
|  | | Pastoralist | | | | | Petty trade | | Government employee | | | | | Total | | | |  | | | |  |
|  | | Number | | | % | | Number | % | Number | | | | % | Number | | | % |  | | | |  |
| Male headed, with a wife or wives | | 76 | | | 49.35 | | 2 | 1.30 | 5 | | | | 3.25 | 110 | | | 71.43 |  | | | |  |
| Male headed, divorced, single or widowed | | 3 | | | 1.95 | | 1 | 0.65 | 1 | | | | 0.65 | 9 | | | 5.84 |  | | | |  |
| Female headed, divorced, single or widowed | | 12 | | | 7.79 | | 0 | 0.00 | 1 | | | | 0.65 | 16 | | | 10.39 |  | | | |  |
| Female headed, huSSBand away, wife makes most household/agricultural decisions, | | 7 | | | 4.55 | | 0 | 0.00 | 1 | | | | 0.65 | 13 | | | 8.44 |  | | | |  |
| Child headed (age 18 years or under)/Orphan | | 1 | | | 0.65 | | 0 | 0.00 | 0 | | | | 0.00 | 2 | | | 1.30 |  | | | |  |
| Others | | 1 | | | 0.65 | | 0 | 0.00 | 0 | | | | 0.00 | 2 | | | 1.30 |  | | | |  |
| Total | | 100 | | | 64.94 | | 3 | 1.95 | 8 | | | | 5.19 | 152 | | | 98.70 |  | | | |  |
| Topographic preference of *P.juliflora* | | | | | | | | | | | | | | | | | | | | | |  |
|  | | |  | |  | |  |  |  | | | |  | |  | |  |  | | | |  |
|  | | | Hilly areas | | | | Flat lands | | Total | | | | | |  | |  |  | | | |  |
|  | | | Number | | % | | Number | % | Number | | | | % | |  | |  |  | | | |  |
| Male headed, with a wife or wives | | | 1 | | 0.65 | | 89 | 57.79 | 110 | | | | 71.43 | |  | |  |  | | | |  |
| Male headed, divorced, single or widowed | | | 0 | | 0.00 | | 7 | 4.55 | 9 | | | | 5.84 | |  | |  |  | | | |  |
| Female headed, divorced, single or widowed | | | 1 | | 0.65 | | 11 | 7.14 | 16 | | | | 10.39 | |  | |  |  | | | |  |
| Female headed, huSSBand away, wife makes most household/agricultural decisions, | | | 0 | | 0.00 | | 11 | 7.14 | 13 | | | | 8.44 | |  | |  |  | | | |  |
| Child headed (age 18 years or under)/Orphan | | | 0 | | 0.00 | | 2 | 1.30 | 2 | | | | 1.30 | |  | |  |  | | | |  |
| Others | | | 0 | | 0.00 | | 2 | 1.30 | 2 | | | | 1.30 | |  | |  |  | | | |  |
| Total | | | 2 | | 1.30 | | 122 | 79.22 | 152 | | | | 98.70 | |  | |  |  | | | |  |
|  | | | How do think *P.juliflora* dispersed? | | | | | |  | | | |  | |  | |  |  | | | |  |
|  | | | Self | | | | Livestock | | Total | | | | | |  | |  |  | | | |  |
|  | | | Number | | % | | Number | % | Number | | | | % | |  | |  |  | | | |  |
| Male headed, with a wife or wives | | | 8 | | 5.19 | | 100 | 64.94 | 110 | | | | 71.43 | |  | |  |  | | | |  |
| Male headed, divorced, single or widowed | | | 1 | | 0.65 | | 8 | 5.19 | 9 | | | | 5.84 | |  | |  |  | | | |  |
| Female headed, divorced, single or widowed | | | 1 | | 0.65 | | 15 | 9.74 | 16 | | | | 10.39 | |  | |  |  | | | |  |
| Female headed, huSSBand away, wife makes most household/agricultural decisions, | | | 0 | | 0.00 | | 13 | 8.44 | 13 | | | | 8.44 | |  | |  |  | | | |  |
| Child headed (age 18 years or under)/Orphan | | | 1 | | 0.65 | | 1 | 0.65 | 2 | | | | 1.30 | |  | |  |  | | | |  |
| Others | | | 0 | | 0.00 | | 2 | 1.30 | 2 | | | | 1.30 | |  | |  |  | | | |  |
| Total | | | 11 | | 7.14 | | 139 | 90.26 | 152 | | | | 98.70 | |  | |  |  | | | |  |
|  | | | Wild animal most dispersed *P.juliflora*? | | | | | |  | | | |  | |  | |  |  | | | |  |
|  | | | Warthog | | | | Gazelle |  | Dik-dik | | | |  | | Ape | |  | Total | | | |  |
|  | | | Number | | % | | Number | % | Number | | | | % | | Number | | % | Number | % | | |  |
| Male headed, with a wife or wives | | | 80 | | 51.95 | | 0 | 0.00 | 0 | | | | 0.00 | | 1 | | 0.65 | 110 | 71.43 | | |  |
| Male headed, divorced, single or widowed | | | 6 | | 3.90 | | 0 | 0.00 | 1 | | | | 0.65 | | 0 | | 0.00 | 9 | 5.84 | | |  |
| Female headed, divorced, single or widowed | | | 9 | | 5.84 | | 1 | 0.65 | 0 | | | | 0.00 | | 0 | | 0.00 | 16 | 10.39 | | |  |
| Female headed, huSSBand away, wife makes most household/agricultural decisions, | | | 8 | | 5.19 | | 0 | 0.00 | 0 | | | | 0.00 | | 0 | | 0.00 | 13 | 8.44 | | |  |
| Child headed (age 18 years or under)/Orphan | | | 1 | | 0.65 | | 1 | 0.65 | 0 | | | | 0.00 | | 0 | | 0.00 | 2 | 1.30 | | |  |
| Others | | | 2 | | 1.30 | | 0 | 0.00 | 0 | | | | 0.00 | | 0 | | 0.00 | 2 | 1.30 | | |  |
| Total | | | 106 | | 68.83 | | 2 | 1.30 | 1 | | | | 0.65 | | 1 | | 0.65 | 152 | 98.70 | | |  |
|  | | | Negative effects of *P.juliflora* of livestock? | | | | | | | | | |  | |  | |  |  |  | | |  |
|  | | | Animals get injured | | | Animals die from eating | | | | poisoning/toxic effects | | | | | Total | |  |  |  | | |  |
|  | | | Number | | % | Number | | % | | Number | | | % | | Number | | % |  |  | | |  |
| Male headed, with a wife or wives | | | 40 | | 25.97 | 22 | | 14.29 | | 33 | | | 21.43 | | 109 | | 70.78 |  |  | | |  |
| Male headed, divorced, single or widowed | | | 3 | | 1.95 | 2 | | 1.30 | | 0 | | | 0.00 | | 6 | | 3.90 |  |  | | |  |
| Female headed, divorced, single or widowed | | | 9 | | 5.84 | 3 | | 1.95 | | 3 | | | 1.95 | | 16 | | 10.39 |  |  | | |  |
| Female headed, huSSBand away, wife makes most household/agricultural decisions, | | | 5 | | 3.25 | 4 | | 2.60 | | 1 | | | 0.65 | | 13 | | 8.44 |  |  | | |  |
| Child headed (age 18 years or under)/Orphan | | | 1 | | 0.65 | 0 | | 0.00 | | 1 | | | 0.65 | | 2 | | 1.30 |  |  | | |  |
| Others | | | 2 | | 1.30 | 0 | | 0.00 | | 0 | | | 0.00 | | 2 | | 1.30 |  |  | | |  |
| Total | | | 60 | | 38.96 | 31 | | 20.13 | | 38 | | | 24.68 | | 148 | | 96.10 |  |  | | |  |
| Is spread *P.juliflora* increasing or declining? | | | | | | | | | | | | | | | | | | | | | |  |
|  | | | | Yes | | | No | | | |  | |  |  | | |  |  | | |  |  |
|  | | | | Number | % | | Number | % | | |  | |  |  | | |  |  | | |  |  |
| Male headed, with a wife or wives | | | | 105 | 68.18 | | 4 | 2.60 | | |  | |  |  | | |  |  | | |  |  |
| Male headed, divorced, single or widowed | | | | 6 | 3.90 | | 0 | 0.00 | | |  | |  |  | | |  |  | | |  |  |
| Female headed, divorced, single or widowed | | | | 16 | 10.39 | | 0 | 0.00 | | |  | |  |  | | |  |  | | |  |  |
| Female headed, huSSBand away, wife makes most household/agricultural decisions, | | | | 13 | 8.44 | | 0 | 0.00 | | |  | |  |  | | |  |  | | |  |  |
| Child headed (age 18 years or under)/Orphan | | | | 2 | 1.30 | | 0 | 0.00 | | |  | |  |  | | |  |  | | |  |  |
| Others | | | | 2 | 1.30 | | 0 | 0.00 | | |  | |  |  | | |  |  | | |  |  |
| Total | | | | 144 | 93.51 | | 4 | 2.60 | | |  | |  |  | | |  |  | | |  |  |
|  | | | | Which part eaten *P.juliflora* by donkeys? | | | | | | | | |  |  | | |  |  | | |  |  |
|  | | | | Pods | | | Leaf | | | Total | | | |  | | |  |  | | |  |  |
|  | | | | Number | % | | Number | % | | Number | | | % |  | | |  |  | | |  |  |
| Male headed, with a wife or wives | | | | 106 | 68.83 | | 0 | 0.00 | | 109 | | | 70.78 |  | | |  |  | | |  |  |
| Male headed, divorced, single or widowed | | | | 6 | 3.90 | | 0 | 0.00 | | 6 | | | 3.90 |  | | |  |  | | |  |  |
| Female headed, divorced, single or widowed | | | | 16 | 10.39 | | 0 | 0.00 | | 16 | | | 10.39 |  | | |  |  | | |  |  |
| Female headed, huSSBand away, wife makes most household/agricultural decisions, | | | | 11 | 7.14 | | 1 | 0.65 | | 13 | | | 8.44 |  | | |  |  | | |  |  |
| Child headed (age 18 years or under)/Orphan | | | | 2 | 1.30 | | 0 | 0.00 | | 2 | | | 1.30 |  | | |  |  | | |  |  |
| Others | | | | 2 | 1.30 | | 0 | 0.00 | | 2 | | | 1.30 |  | | |  |  | | |  |  |
| Total | | | | 143 | 92.86 | | 1 | 0.65 | | 148 | | | 96.10 |  | | |  |  | | |  |  |

| Which measures are taken to control *P.juliflora*? | | | | | | | | | | | | | | | | | | |
| --- | --- | --- | --- | --- | --- | --- | --- | --- | --- | --- | --- | --- | --- | --- | --- | --- | --- | --- |
|  | MU | | MCCMS | | MCUJS | | Fire | | Chemical | | CSD | | UbD | |  | Total | | |
|  | Number | % | Number | % | Number | % | Number | % | Number | % | Number | % | Number | % |  | Number | | % |
| Male headed with wife | 19 | 12.34 | 13 | 8.44 | 1 | 0.65 | 55 | 35.71 | 10 | 6.49 | 1 | 0.65 | 1 | 0.65 |  | 109 |  | 70.78 |
| Male headed, divorced | 1 | 0.65 | 1 | 0.65 | 0 | 0.00 | 3 | 1.95 | 1 | 0.65 | 0 | 0.00 | 0 | 0.00 |  | 6 |  | 3.90 |
| Female headed, divorced | 2 | 1.30 | 1 | 0.65 | 0 | 0.00 | 8 | 5.19 | 3 | 1.95 | 0 | 0.00 | 0 | 0.00 |  | 16 |  | 10.39 |
| Female headed, huSSBand away | 5 | 3.25 | 1 | 0.65 | 0 | 0.00 | 1 | 0.65 | 5 | 3.25 | 0 | 0.00 | 0 | 0.00 |  | 13 |  | 8.44 |
| Child headed | 0 | 0.00 | 1 | 0.65 | 0 | 0.00 | 1 | 0.65 | 0 | 0.00 | 0 | 0.00 | 0 | 0.00 |  | 2 |  | 1.30 |
| Others | 1 | 0.65 | 0 | 0.00 | 0 | 0.00 | 1 | 0.65 | 0 | 0.00 | 0 | 0.00 | 0 | 0.00 |  | 2 |  | 1.30 |
| Total | 28 | 18.18 | 17 | 11.04 | 1 | 0.65 | 69 | 44.81 | 19 | 12.34 | 1 | 0.65 | 1 | 0.65 |  | 148 |  | 96.10 |

Positions in Kebeles

|  | | Do you have position in your Kebele? | | | | | | |  | |  | |  | |  | |  | | |  | |  | |
| --- | --- | --- | --- | --- | --- | --- | --- | --- | --- | --- | --- | --- | --- | --- | --- | --- | --- | --- | --- | --- | --- | --- | --- |
|  | | Why was *P.juliflora* introduced in your site? | | | | | | | | | | |  | |  | |  | | |  | |  | |
|  | | Fuel wood purposes | | | | | | Shade purposes | | | Shelterbelts | | | | other | |  | | | Total | |  | |
|  | | Number | | | % | | | Number | % | | Number | | % | | Number | | % | | | Number | | % | |
| Yes | | 48 | | | 31.17 | | | 29 | 18.83 | | 2 | | 1.30 | | 24 | | 15.58 | | | 111 | | 72.08 | |
| No | | 14 | | | 9.09 | | | 6 | 3.90 | | 2 | | 1.30 | | 6 | | 3.90 | | | 40 | | 25.97 | |
| Total | | 62 | | | 40.26 | | | 35 | 22.73 | | 4 | | 2.60 | | 30 | | 19.48 | | | 151 | | 98.05 | |
| Preferred site for *P.juliflora* establishment | | | | | | | | | | | | | | | | | | | | | | |  |
|  | Homestead | | | Roadsides | | | AMF | | | | | Along rivers | | | | In Wood lands | | | Total | |  | |  |
|  | Frequency | | % | Frequency | | % | Frequency | | | % | | Frequency | | % | | Frequency | | % | Frequency | | % | |  |
| Yes | 28 | | 18.18 | 12 | | 7.79 | 20 | | | 12.99 | | 1 | | 0.65 | | 1 | | 0.65 | 111 | | 72.08 | |  |
| No | 11 | | 7.14 | 8 | | 5.19 | 6 | | | 3.90 | | 0 | | 0.00 | | 0 | | 0.00 | 40 | | 25.97 | |  |
| Total | 39 | | 25.32 | 20 | | 12.99 | 26 | | | 16.88 | | 1 | | 0.65 | | 1 | | 0.65 | 151 | | 98.05 | |  |

| What benefits you get *from P.juliflora*? | | | | | | | | | | | | | | | | | | | | |
| --- | --- | --- | --- | --- | --- | --- | --- | --- | --- | --- | --- | --- | --- | --- | --- | --- | --- | --- | --- | --- |
|  | SfLiv | | Shb | | HC | | CfF | | SWC | | AE | | SSB |  | Fodder |  | others |  | Total |  |
|  | Number | % | Number | % | Number | % | Number | % | Number | % | Number | % | Number | % | Number | % | Number | % | Number | % |
| Yes | 14 | 9.09 | 4 | 2.60 | 7 | 4.55 | 5 | 3.25 | 1 | 0.65 | 1 | 0.65 | 0 | 0.00 | 1 | 0.65 | 1 | 0.65 | 111 | 72.08 |
| No | 6 | 3.90 | 2 | 1.30 | 2 | 1.30 | 5 | 3.25 | 1 | 0.65 | 0 | 0.00 | 1 | 0.65 | 1 | 0.65 | 5 | 3.25 | 40 | 25.97 |
| Total | 20 | 12.99 | 6 | 3.90 | 9 | 5.84 | 10 | 6.49 | 2 | 1.30 | 1 | 0.65 | 1 | 0.65 | 2 | 1.30 | 6 | 3.90 | 151 | 98.05 |

|  | | What is your position in your kebele? | | | | | | | | | | |  | |  | | |  | |  | | |  |  | |  |  |  |
| --- | --- | --- | --- | --- | --- | --- | --- | --- | --- | --- | --- | --- | --- | --- | --- | --- | --- | --- | --- | --- | --- | --- | --- | --- | --- | --- | --- | --- |
| If yes number of coppices | | | | | | | | | | | | | | | | | | | | | | | | | | | | |
|  | | | 0 | |  | 1 | |  | | 6 | |  | | | 7 | |  | | | 20 |  | | | 25 | |  | Total |  |
|  | | | Number | | % | Number | | % | | Number | | % | | | Number | | % | | | Number | % | | | Number | | % | Number | % |
| NPoKM | | | 92 | | 59.74 | 1 | | 0.65 | | 1 | | 0.65 | | | 1 | | 0.65 | | | 1 | 0.65 | | | 1 | | 0.65 | 109 | 70.78 |
| Chairperson | | | 8 | | 5.19 | 0 | | 0.00 | | 0 | | 0.00 | | | 0 | | 0.00 | | | 0 | 0.00 | | | 0 | | 0.00 | 8 | 5.19 |
| secretory | | | 0 | | 0.00 | 0 | | 0.00 | | 0 | | 0.00 | | | 0 | | 0.00 | | | 0 | 0.00 | | | 0 | | 0.00 | 1 | 0.65 |
| Youth | | | 1 | | 0.65 | 0 | | 0.00 | | 0 | | 0.00 | | | 0 | | 0.00 | | | 0 | 0.00 | | | 0 | | 0.00 | 1 | 0.65 |
| Committee member | | | 14 | | 9.09 | 0 | | 0.00 | | 1 | | 0.65 | | | 0 | | 0.00 | | | 0 | 0.00 | | | 0 | | 0.00 | 15 | 9.74 |
| member | | | 17 | | 11.04 | 0 | | 0.00 | | 0 | | 0.00 | | | 0 | | 0.00 | | | 0 | 0.00 | | | 0 | | 0.00 | 17 | 11.04 |
| Total | | | 132 | | 85.71 | 1 | | 0.65 | | 2 | | 1.30 | | | 1 | | 0.65 | | | 1 | 0.65 | | | 1 | | 0.65 | 151 | 98.05 |
| Negative effects *P.juliflora* on rangeland? | | | | | | | | | | | | | | | | | | | | | | | |  |  |  |  |  |
|  | | | TWA | | | TN | | SSL | | Total | | |  | |  | | |  | | |  | | |  |  |  |  |  |
|  | | | Number | | | % | | Number | | % | | | Number | | % | | | Number | | | % | | |  |  |  |  |  |
| NPoKM | | | 43 | | | 27.92 | | 17 | | 11.04 | | | 0 | | 0.00 | | | 104 | | | 67.53 | | |  |  |  |  |  |
| Chairperson | | | 4 | | | 2.60 | | 2 | | 1.30 | | | 1 | | 0.65 | | | 8 | | | 5.19 | | |  |  |  |  |  |
| secretory | | | 1 | | | 0.65 | | 0 | | 0.00 | | | 0 | | 0.00 | | | 1 | | | 0.65 | | |  |  |  |  |  |
| Youth | | | 0 | | | 0.00 | | 1 | | 0.65 | | | 0 | | 0.00 | | | 1 | | | 0.65 | | |  |  |  |  |  |
| Committee member | | | 3 | | | 1.95 | | 4 | | 2.60 | | | 0 | | 0.00 | | | 15 | | | 9.74 | | |  |  |  |  |  |
| member | | | 14 | | | 9.09 | | 3 | | 1.95 | | | 0 | | 0.00 | | | 18 | | | 11.69 | | |  |  |  |  |  |
| Total | | | 65 | | | 42.21 | | 27 | | 17.53 | | | 1 | | 0.65 | | | 147 | | | 95.45 | | |  |  |  |  |  |

| HOUSEHOLD relationships | | | | | | | | | | | | | | |
| --- | --- | --- | --- | --- | --- | --- | --- | --- | --- | --- | --- | --- | --- | --- |
|  | If yes number of coppices | | | |  |  |  |  |  |  |  |  |  |  |
|  | 0 |  | 1 |  | 6 |  | 7 |  | 20 |  | 25 |  | Total |  |
|  | Number | % | Number | % | Number | % | Number | % | Number | % | Number | % | Number | % |
| Head | 106 | 68.83 | 1 | 0.65 | 1 | 0.65 | 1 | 0.65 | 0 | 0 | 0 | 0 | 118 | 76.62 |
| Spouse | 1 | 0.65 | 0 | 0.00 | 0 | 0.00 | 0 | 0.00 | 0 | 0 | 0 | 0 | 1 | 0.65 |
| Parent | 10 | 6.49 | 0 | 0.00 | 0 | 0.00 | 0 | 0.00 | 0 | 0 | 1 | 0.65 | 13 | 8.44 |
| Child | 8 | 5.19 | 0 | 0.00 | 0 | 0.00 | 0 | 0.00 | 0 | 0 | 0 | 0 | 9 | 5.84 |
| Nephew/Niece | 2 | 1.30 | 0 | 0.00 | 0 | 0.00 | 0 | 0.00 | 0 | 0 | 0 | 0 | 2 | 1.30 |
| Son/daughter-in-law | 1 | 0.65 | 0 | 0.00 | 0 | 0.00 | 0 | 0.00 | 0 | 0.00 | 0 | 0 | 1 | 0.65 |
| Brother/sister | 4 | 2.60 | 0 | 0.00 | 1 | 0.65 | 0 | 0.00 | 1 | 0.65 | 0 | 0 | 7 | 4.55 |
| Total | 132 | 85.71 | 1 | 0.65 | 2 | 1.30 | 1 | 0.65 | 1 | 0.65 | 1 | 0.65 | 151 | 98.05 |

| Notices: DAP is development agro-pastoralist, TWA is it takes water away, TN is it takes nutrients, SSL is share sun light, NPoKM is none position of kebele member, Notice: AMF is around mechanized farming, SfLiv is shade for livestock, Shb is shade for human beings, HC is house construction, CfF is cutting for fencing, SWC is soil and water conservation, SSB is shelter belt, AE is Ameliorating effects, MU is manage by utilization, MCCMS is mechanical control by cutting mature stems, MCUJS is mechanical control by uprooting juvenile stems, CSD is control seed dispersal, UbD is uprooting by dozer, AMF is around mechanized farming |
| --- |

## Appendix 29. Income from livelihood options in South Afar Region

| Income (Birr/year) | | | | | | | | | | | |
| --- | --- | --- | --- | --- | --- | --- | --- | --- | --- | --- | --- |
| Variables | Statistics | Milk | Cattle | labour | Vegetables | Fruits | Crops | WPPJ | Charcoal PJ | Petty trade | WPNSp |
| DN | *χ^2^* | 5.8 | 0.1 | 0.0 | 35.7 | 54.9 | 43.1 | 0.2 | 35.3 | 0.6 | 0.0 |
|  | df | 1 | 1 | 1 | 1 | 1 | 1 | 1 | 1 | 1 | 1 |
|  | *P-value* | 0.016 | 0.735 | 0.861 | <0.0001 | <0.0001 | <0.0001 | 0.655 | <0.0001 | 0.432 | 0.950 |
| Site | *χ^2^* | 46.1 | 44.5 | 32.7 | 79.0 | 101.1 | 76.6 | 1.6 | 72.2 | 3.8 | 0.7 |
|  | df | 3 | 3 | 3 | 3 | 3 | 3 | 3 | 3 | 3 | 3 |
|  | *P-value* | <0.0001 | <0.0001 | <0.0001 | <0.0001 | <0.0001 | <0.0001 | 0.662 | <0.0001 | 0.286 | 0.879 |
| Sex | *χ^2^* | 0.6 | 2.1 | 2.6 | 2.6 | 0.7 | 1.7 | 0.8 | 4.1 | <0.0001 | <0.0001 |
|  | df | 1 | 1 | 1 | 1 | 1 | 1 | 1 | 1 | 1 | 1 |
|  | *P-value* | 0.424 | 0.150 | 0.106 | 0.110 | 0.404 | 0.187 | 0.359 | 0.043 | 0.832 | 0.882 |
| Education | *χ^2^* | 7.4 | 5.3 | 7.9 | 14.5 | 14.8 | 8.6 | 1.0 | 3.5 | 6.9 | 1.2 |
|  | df | 3 | 3 | 3 | 3 | 3 | 3 | 3 | 3 | 3 | 3 |
|  | *P-value* | 0.061 | 0.154 | 0.049 | 0.002 | 0.002 | 0.036 | 0.804 | 0.317 | 0.075 | 0.759 |
| Wealth | *χ^2^* | 1.0 | 9.0 | 2.5 | 12.6 | 5.8 | 7.7 | 0.3 | 8.4 | 0.6 | 1.9 |
|  | df | 2 | 2 | 2 | 2 | 2 | 2 | 2 | 2 | 2 | 2 |
|  | *P-value* | 0.61 | 0.01 | 0.29 | <0.0001 | 0.05 | 0.02 | 0.84 | 0.02 | 0.75 | 0.40 |

Notices:DN is district df is degree of freedom, WPPJ is woody products from *P.juliflora,* PJ is *P.juliflora,* WPNSp is woody products from native species
