# Supplementary material for: Community perceptions towards invasion of Prosopis juliflora, utilization, and its control options in Afar region, Northeast Ethiopia
Source: PLoS One. 2022 Jan 25;17(1):e0261838. doi: 10.1371/journal.pone.0261838 (PMC8789103; doi:10.1371/journal.pone.0261838)
Supplement: Attachment — Submitted filename: Answer.docx [file pone.0261838.s004.docx]

Answer

Journal: PLOS ONE

Manuscript ID: PONE-D-20-29179

Editor concerns: There are various serious concerns: i) the analysis conducted with the data: a serious pitfall in transforming categorial data before undertaking non-parametric analysis; the results, interpretation and conclusion are not therefore convincing; ii) lots of unecessary details are given in the results and the discussion sections which clearly impede the focus of the message; iii) the manuscript needs a thorough editing to improve readability and grammatical oddities that are maintained throughout.

Answer: We tried to edit and reduce the errors; the manuscript is edited to correct grammatical errors and its readability

Reviewer #1

Concern: (1) You noted that the data were not normally distributed which is probably correct. Most of the input data are in fact categorical and should not be transformed. The data were then transformed before being analyzed using non-parametric methods. The transformation is inappropriate and may have affected your results. The data must be analyzed using methods designed for such data

Answer: The data now analyzed using methods designed for data analysis before transformation

Concern (2): You only mention the SPSS package but not the actual routines/tools/methods used to do the analysis. It is essential that you are as explicit as possible in describing what you did because I need to know that you used an appropriate tool/method. The results and the discussion sections are very lengthy and loaded with detail which would be of little interest to most of the prospective readers (and is generally evident in the figures). Try to reduce them to the essentials and try to focus on exploring the reasons why people may have given the responses they did and why there were differences between the districts that you sampled. The more succinct you can be the more likely it is that people will read and cite your paper.

Answer

Now we tried to mention the appropriate methods used for the analysis

The length section of results and discussions are reduced

Reviewer #2

Concerns

1. It has to be acknowledged that the method used in this study need some clarification;

Answer: now it is clear

2. The writing style in all sections of the paper need to be revised;

Revised

3. The introduction must be restructured to clearly show and demonstrate the relevance and importance of your study as well as its added value to the wealth of knowledge on the same subject;

Answer: Improved

4. The data analysis is largely descriptive;

Yes the nature of the title
5. The section “results” is so long and not straightforward;

Answer: reduced
Specific comments are well taken and the document is improved
